# Consistency-based detection of potential tumor-specific deletions in matched normal/tumor genomes

**DOI:** 10.1186/1471-2105-12-S9-S21

**Published:** 2011-10-05

**Authors:** Roland Wittler, Cedric Chauve

**Affiliations:** 1 Department of Mathematics, Simon Fraser University, Burnaby, BC, V5A 1S6, Canada; 2 Technische Fakultät, Universität Bielefeld, Bielefeld, 33594, Germany

## Abstract

**Background:**

Structural variations in human genomes, such as insertions, deletion, or rearrangements, play an important role in cancer development. Next-Generation Sequencing technologies have been central in providing ways to detect such variations. Most existing methods however are limited to the analysis of a single genome, and it is only recently that the comparison of closely related genomes has been considered. In particular, a few recent works considered the analysis of data sets obtained by sequencing both tumor and healthy tissues of the same cancer patient. In that context, the goal is to detect variations that are specific to exactly one of the genomes, for example to differentiate between patient-specific and tumor-specific variations. This is a difficult task, especially when facing the additional challenge of the possible contamination of healthy tissues by tumor cells and conversely.

**Results:**

In the current work, we analyzed a data set of paired-end short-reads, obtained by sequencing tumor tissues and healthy tissues, both from the same cancer patient. Based on a combinatorial notion of conflict between deletions, we show that in the tumor data, more deletions are predicted than there could actually be in a diploid genome. In contrast, the predictions for the data from normal tissues are almost conflict-free. We designed and applied a method, specific to the analysis of such pooled and contaminated data sets, to detect potential tumor-specific deletions. Our method takes the deletion calls from both data sets and assigns reads from the mixed tumor/normal data to the normal one with the goal to minimize the number of reads that need to be discarded to obtain a set of conflict-free deletion clusters. We observed that, on the specific data set we analyze, only a very small fraction of the reads needs to be discarded to obtain a set of consistent deletions.

**Conclusions:**

We present a framework based on a rigorous definition of consistency between deletions and the assumption that the tumor sample also contains normal cells. A combined analysis of both data sets based on this model allowed a consistent explanation of almost all data, providing a detailed picture of candidate patient- and tumor-specific deletions.

## Background

A fundamental goal of human genomics is to identify and describe differences among human genomes. Besides the detection of single nucleotide polymorphisms, larger mutation events, such as deletions, insertions, inversions, or inter-chromosomal rearrangements, can have a crucial impact on genomes function. They can for instance result in loss, mutation or fusion of genes that can be linked to certain diseases such as cancer. The characterization of structural variations can thus help shed some light on the complex mechanisms in cancer biology [1-4].

### Structural variations discovery

Current sequencing technologies enable fast sequencing of human genomes at high coverage and low cost. Usually, multiple copies of a genome are randomly broken into small fragments which are then sequenced. Most of these techniques allow to read DNA fragments from both sides, resulting in a large set of *paired-end reads.* Saving the time and cost intensive assembly and finishing steps which would be necessary to determine the full genome sequence, the read pairs can directly be used to detect structural variations by *paired-end mapping.* The paired-end reads from a newly sequenced *donor genome* are mapped onto a *reference genome* which is already assembled to a complete DNA-sequence [5,6]. In a region where the two genomes do not differ, the mapped reads have the correct orientation and their distance coincides with the fragment length in the donor genome. Such a mapping is called *concordant.* However, if a mapping is *discordant*, i.e. the orientation is incorrect or the distance differs significantly from the expected fragment length, this indicates a putative structural variation in the donor w.r.t. the reference. This concept was fist introduced by Volik et al. [7] and Tuzun et al. [8]. In recent years, many tools have been developed that follow this approach to identify putative structural variations (reviewed in [9-11]).

Another approach is to actually assemble all reads to obtain the exact sequence. To avoid the computational expense of full *de novo* assembly [12], one can restrict the process to only reads from regions suspect to harbor a variation, as for instance done in [13].

### Tumor genomes analysis

Besides problems in the accurate prediction of variations due to ambiguous mappings, mappings in repeat regions etc. [6], these approaches have a fundamental shortcoming for the analysis of cancer data: They do not differentiate between inherent, patient-specific variations and those which are *tumor-specific.* Even if the data of both a tumor sample and a normal sample (i.e. from a tissue not affected by cancer) from the same individual is available, this is not a trivial task [3,4,14-16]. In particular, considering any discordant mapping from the tumor data overlapping a structural variation found in the normal data as non-tumor-specific could result in missing tumor-specific variations since different structural variations can be overlapping or very closely located. Another difficulty in analyzing cancer data is that a cancer sample is most likely a *mixed* sample: Although taken from tumor tissue, it usually contains also normal cells [2,6,17]. Hence, we have to tackle the "need to simultaneously analyze data from tumor and patient-matched normal tissue … and the ability to handle samples with unknown levels of non-tumor contamination" [17].

To our knowledge, very few methods allow a combined analysis of pooled data sets, such as a normal and a tumor sample, to detect deletions of arbitrary length indicated by discordant mappings. BreakDancer [18] was used in [16], although it was not designed explicitly for such a task. In [19], it was proposed to cluster together only mappings from the tumor data set which do not overlap any discordant mapping from the normal data set. This might result in discarding tumor-specific variations that overlap or are close to normal variations, a problem we address in the present work. They also suggest pooling the mappings obtained from both samples into one data set and to assemble them into clusters calling for the same structural variations. Clusters containing no mappings from the normal data set are then considered being tumor-specific. Recently, Hormozdiari et al. [20] introduced an approach to detect common variations among several genomes (The program described in [20] was not publicly available at the time of submission). However, their method focuses on ambiguously mapped read pairs and does not consider concordant mapped pairs. Other methods that consider pooled data sets are for analyzing only small scale deletions of a few base pairs in size [14,21,22].

### Contribution

The goal of the present paper is to address a very specific problem in the analysis of matched tumor/normal genomes: the detection of putative tumor-specific deletions that overlap or are closely located to normal deletions.

The motivation for this question stems from the preliminary analysis of data from an adenocarcinoma patient [23], obtained by sequencing both normal cells and tumor cells. We found that, for the tumor data, many overlapping deletions were called. Moreover we observed that, in some regions, more deletions were called than there could actually be on a diploid genome. Especially in regions where the normal data indicate a possible deletion, we found a high signal of inconsistency for the deletions in the tumor sample, whereas in the normal data this signal was very low.

To detect such regions, we rely on a combinatorial notion of *conflicting deletions* that represents deletions, defined by clusters of discordant mappings which overlap significantly and could then not happen together on the same diploid genome. The notion of conflicting structural variations was introduced in [24] to handle in particular reads that can be mapped to several locations. We extend the work of [24] by describing a rigorous definition of conflicts that accounts for the difference between deletions and breakpoint region, and by showing that conflicts in an haploid genome are limited to sets of two or three deletion clusters.

In order to extract from these data putative tumor-specific deletions that would be consistent with the conflict-free deletions detected in the normal sample, we designed a method based on the combined analysis of both the tumor and normal data. Our approach assumes a mixture of normal and cancer cells in the tumor sample, i.e., four copies of each chromosome (two from diploid normal genomes and two from diploid tumor genome). Our combinatorial objective is to find a partition of the mappings from the tumor data set into four sets (each one assigned to one specific chromosome copy) that corresponds to a consistent set of deletions. If not all mappings can be incorporated into a consistent deletion call, we aim at minimizing the number of mappings required to be discarded to reach consistency. This approach allowed us to consistently explain most of the data we analyzed: very few mappings needed to be discarded to be able to both refine normal deletions and detect putative tumor-specific deletions that were both conflict-free and consistent with the normal deletions. An implementation of our method can be found in Additional file 1.

## Methods

### Mappings and deletion clusters

We first recall the standard combinatorial approach to detect deletions in a set of mapped paired-end short-reads, based on clustering together pairs of reads that indicate a similar deletion. We assume here that the sequenced short reads were first mapped onto the reference genome, resulting in a unique location for each read; several tools exist for this task, including some that can handle reads that map in several locations of the reference genome [24,25].

**Figure 1:**
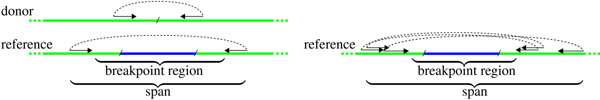
**Mappings**. Illustration of (left) a mapping and (right) a valid cluster.

A deletion is a segment of the reference genome that is not present in the donor genome. The precise location of the deletion in the donor genome is called its *breakpoint.* A pair of reads spanning the breakpoint in the donor genome will be mapped to the reference with a distance increased by the size of the deletion, and with proper orientation of its two reads. For such a mapping m, let *span*(*m*) be the interval from the left end of the left read to the right end of the right read. We assume the size range (including the reads) of the sequenced fragments (called the insert size) is known and comprised between minLen (at least) and maxLen (at most). A deletion has then to be of size at least *delMin*(*m*) := |*span*(*m*)| – maxLen and at most *delMax*(*m*) := |*span*(*m*)| – minLen. Obviously, the deleted segment has to be between the right end of the left read and the left end of the right read—we call this the *breakpoint region*, *br*(*m*), of the mapping. See Figure 1(left) for an illustration. Note that the approach described above does not aim at detecting very small deletions (typically of size 10 bases or less), which can be done by analyzing directly the read mappings [21,22].

If the coverage of the sequenced genome by the reads is high, one can expect that a deletion breakpoint is covered by several pairs of reads. A large number of mappings indicating the same deletion increases naturally the confidence in this potential deletion. Furthermore, the specification of the deletion, its location and length, will generally be more precise if it is based on more observations. Therefore, it is common to cluster mappings that can be explained by the same event. More precisely, in the case of a deletion, a set *c* = {*m*_1_,…, *m_n_*} is a *valid* cluster of mappings if there is a possible deletion length compatible with all mappings. Let  and  . Then there exists delLen ≤ |*br*(*c*)| such that for each *m* ∈ *c* : *delMin*(*m*) ≤ delLen ≤ *delMax*(*m*)*.* For a valid cluster *c*, we can infer the size range of the deletion:

*delMin*(*c*) := max {*delMin*(*m*) | *m* ∈ *c*} and

*delMax*(*c*) := min {*delMax*(*m*) | *m* ∈ *c*} .

A cluster *c* is called *maximal* if it is valid and there is no mapping *m* ∉ *c* such that *c* ∪ {*m*} is valid. Figure 1(right) shows an illustration of a cluster.

### Haploid conflicts

To introduce the notion of conflict, we first consider that all deletions may have taken place on a haploid genome. We discuss the diploid case in the next section.

**Figure 2:**
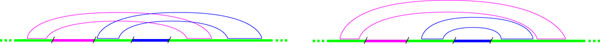
**Overlapping clusters.** We assume that each cluster describes exactly one deletion. Therefore, clusters overlapping as shown in this figure are not consistent. Only the surrounding shape of the mappings in the cluster, and the position and size of the deleted segments are indicated.

The notion of conflicting clusters was introduced in [24] and we refine it here. First, as discussed previously, we assume that a deletion is associated to a cluster (possibly of size 1) of discordant mappings. The deletion associated to a cluster is then located within the breakpoint region of the cluster. Further, if any read—belonging to either a concordant or a discordant pair—is mapped onto a segment in the reference sequence, this segment can obviously not be part of a deletion. Our last assumption is that each deletion is covered by exactly one cluster. Hence, our model does not allow scenarios as shown in Figure 2, and considers such overlapping clusters as conflicting. This approach is motivated by several reasons. First, such configurations, where two overlapping clusters can be neither combined into one valid cluster nor consistently explained by two separate deletions within each cluster, require very specific combinations of fragment length, read length and deletion sizes, and are then quite unlikely to be observed. Also, from a computational point of view, deciding if such overlapping clusters are not conflicting require to investigate splitting the deletion indicated by a cluster into several smaller deletions. As far as we know, this splitting deletion problem has not been considered and there does not seem to be an obvious algorithm to address it. Finally, the additional conflicts stemming from our assumption did not impact the analysis of the data we considered. So disregarding these pathological exceptions leads to a simpler combinatorial model, that proves to be sufficient to explain the data set we analyzed.

**Definition 1. ***Let C be a set of maximal deletion clusters. Then C is* consistent *if for all c* ∈ *C*, *contains an interval of size delMin*(*c*)*. Otherwise it is* conflicting.

If a set of clusters is conflicting, any set containing all these clusters is also conflicting. To detect all conflicts, it is sufficient to find the minimal conflicting sets, a general notion that was used to deal with inconsistency in reconstructing ancestral genomes [26,27].

**Definition 2.*** A set of maximal deletion clusters C is* minimal conflicting *if and only if it is conflicting and*, *for all c* ∈ *C*, *the set C\*{*c*} *is consistent.*

**Lemma 1. ***A minimal conflicting set in a haploid genome contains exactly two or exactly three maximal deletion clusters.*

**Figure 3:**
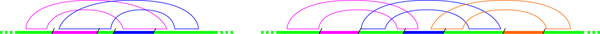
**Minimal Conflicting Sets.** Examples for minimal conflicting sets of two and three deletion clusters.

*Proof.* First, it is not difficult to see that there can be minimal conflicting sets containing exactly two or exactly three maximal clusters (See Figure 3). It then remains to be shown that there is no minimal conflicting set that contains more than three clusters.

To prove this fact, we rely on the following observation: If the breakpoint region of a maximal cluster *c* is covered completely by other maximal clusters *c*_1_, …, *c_k_*, i.e., , then {*c*, *c*_1_,…, *c_k_*} is conflicting.

Now, assume there exists a minimal conflicting set of clusters *C* = {*c*_1_,…, *c_k_*} with *k* > 3 which are all assigned to one chromosome copy. As they are in conflict, they overlap. According to the above observation, if any cluster overlaps with two clusters on one side, these three clusters would create a conflicting set, which contradicts the assumption that *C* is minimal conflicting. Thus, they can only overlap according to a chain structure: Each cluster can overlap with at most one cluster on each side. Without loss of generality, assume that each *c_i_* overlaps with *c_i_*_+1_ for 1 ≤ *i* ≤ *k.*

If *C* is minimal conflicting, {*c*_1_,…, *c_k_*_–1_} is consistent. Since the remaining cluster *c_k_* only overlaps *c_k_*_–1_, there are only two possibilities that result in *C* being conflicting:

Either there is not enough space for the deletion called by *c_k_*_–1_ within its breakpoint region in between the spans of *c_k_*_–2_ and *c_k_*: |*br*(*c_k_*_–1_)\{*span*(*c_k_*_–2_) ∪ *span*(*c_k_*)} | <*delMin*(*c_k_*_–1_). In this case, {*c_k_*_–2_, *c_k_*_–1_, *c_k_*} is conflicting, which contradicts the assumption that *C* is minimal conflicting.

Or, there is not enough space for the deletion called by *c_k_* within its breakpoint region to the right of the end of *c_k_*_–1_: |*br*(*c_k_*\*span*(*c_k_*_–1_)| <*delMin*(*c*k)*.* In this case, {*c_k_*_–1_, *c_k_*} is conflicting, which again contradicts the assumption that *C* is minimal conflicting.

Since concordant mappings define segments in which no variation occurred, they also restrict the space for putative deletions and can thus be involved in conflicts. We can simply extend the above definition of consistency by considering a concordant mapping as a cluster with minimal deletion length zero. Any follow-up definition can be extended analogously.

**Definition 3.***Let C be a set of maximal deletion clusters and M be a set of concordant mappings. Then C and M are* consistent *if for all c* ∈ *C*, *the remaining positions in**contain an interval of size delMin*(*c*), *otherwise they are* conflicting.

In summary, we can identify all haploid conflicts by testing each pair and each triplet of overlapping clusters or mappings. These combinatorial results refine [24], where (1) conflicts were defined only in terms of the length of the breakpoint regions and not of the deletions included in these regions and (2) only conflicts between pairs of clusters were considered.

### Diploid conflicts

A normal human genome is *diploid*, i.e., there are two copies of each chromosome. If a structural variation affects both copies identically, it is *homozygous.* But a variation might also affect only one of the copies, in which case it is *heterozygous.* If a set of clusters is conflicting as defined in the previous section, it is not necessarily conflicting when we consider heterozygous deletions. The conflicting clusters could be assigned to separate chromosome copies and thus be explained by independent, consistent deletions. However, in some cases, even such a separation is impossible. To check consistency for a given set of clusters on a diploid genome, a graph-theoretic approach can be used, that was introduced in [24].

#### A hypergraph model

We define a hypergraph containing each cluster as a node and each minimal conflicting set (as defined in the previous section) as a hyper-edge. A set of clusters is consistent if and only if the corresponding graph is two-colorable, i.e., we can assign one of two colors to each node such that each hyper-edge contains at least one node of each color. In this case, each cluster can be assigned to one of the two chromosome copies such that none of the two copies contains a complete conflicting set. Analogously to the definition of consistency, we can include concordant mappings into this framework by adding a node for each mapping and further edges corresponding to the minimal conflicting sets containing this concordant mapping.

Although this model is a natural and simple way to formally define the consistency problem, it does not provide a simple solution for it right away. In fact, the hypergraph two-colorability problem is known to be NP-hard. The hardness result also holds in our case, where each minimal conflicting set and thus each hyper-edge is of size two or three [28].

#### A heuristic approach to detect diploid conflicts

Therefore, we relied on the following straightforward observation to detect at least a certain type of conflict in polynomial time: A set of three maximal clusters, or two clusters and one concordant mapping, which are pairwise in conflict with respect to one chromosome copy (i.e., they form pairwise haploid conflicts) cannot be conflict-free in a diploid genome. Hence, to get an approximation of the amount of inconsistency in our data, and in particular a lower bound on the number of clusters involved in at least one conflict, we computed all triplets of three clusters, and those containing two clusters and one concordant mapping where the three elements are pairwise in conflict. We are aware that these are not all possible conflicts. Actually, there are other configurations of overlapping clusters causing a diploid conflict which could theoretically exist. However, these configurations require a quite large number of clusters (at least six) overlapping in a very specific way, and we expect these combinations being less likely to occur in real data.

### Analyzing matched normal/tumor data sets

We now turn to our specific problem: Given a deletion cluster detected in the normal data set and one or several deletion clusters detected in the contaminated tumor data set, involved in some conflict, how can we provide, if possible, a consistent explanation of the reads (both concordant and discordant) obtained from this genome region?

In the following, we describe a framework designed to analyze the tumor-subset, whose goal is to provide a set of conflict-free deletion clusters while discarding as few reads as possible.

#### Overlapping component of a normal deletion cluster

To analyze these regions, we define the following subset of the tumor data set, denoted from now as "tumor-subset": First, we collect all discordant mappings from the tumor data set overlapping a normal deletion (more precisely overlapping the breakpoint region of a maximal valid cluster of mappings indicating a deletion). We then iteratively add further deletion mappings which overlap those already added to the subset, which we say are indirectly overlapping the initial normal deletion cluster. We call all mappings which (directly or indirectly) overlap the same normal deletion an *overlapping component.*

In all generality, the overlapping components are not necessarily disjoint and could thus not be treated independently. However, in the considered data set (see Results section) the majority (97.4%) of the overlapping components were disjoint, and we discarded the few remaining components.

As we will see in Results section , the maximal valid clusters in the tumor data and tumor-subset are highly conflicting. When we consider only tumor-specific deletions found by pooling, i.e., a combined processing of the normal and tumor data, the ratio of conflicting clusters increases significantly.

#### A general framework to clear conflicts: assumptions

A central assumption in our approach is that the tumor data originated from a mixed sample containing tumor as well as normal cells, hence two sets of diploid chromosomes. This implies that the read pairs have actually been obtained from four chromosome copies. This assumption is natural for tumor cell sequencing [17]. It is well known that tumor genomes can be affected by ploidy abnormality, such as *loss of heterozygosity* (*LOH*) [*29*] or increased copy number of some chromosomes. Such information can naturally be integrated in our model if known, but they were not available for the data we analyzed, which justified our choice to assume four copies of each chromosome. This proved to be sufficient, as we show later in the Results section.

Next, a deletion can be either *heterozygous* (i.e., it affects only one of the two chromosome copies), or *homozygous* (i.e., the same chromosomal segment is deleted from both copies). In general, deciding if a deletion is heterozygous or homozygous from paired-end mappings is difficult. Probabilistic methods have been designed [30-32], requiring certain parameters, a-priori probabilities or other assumptions. To actually verify the nature of deletions, further experiments, like PCR-analyzes, need to be carried out [33]. Here we assume that all deletions are heterozygous. Our framework could be expanded appropriately by assuming that some deletions are located on all four chromosome copies in the mixed data set. Preliminary investigations showed that this would only slightly increase the number of mappings which cannot be explained by our approach.

#### A general framework to clear conflicts: description

Hence, the problem can be generally described as assigning the largest set of mapping from the tumor data set to one of the four chromosome copies in such a way that no conflict between deletion clusters occur. More precisely, for each overlapping component of a normal deletion cluster *c* and its overlapping mappings *M* = {*m*_1_, …, *m_n_*} in the tumor data set, we solve the following optimization problem.

**Problem 1** (Consistent assignment to four chromosome copies). *Given a set M of mappings and a valid cluster c*, *find a partition of M into disjoint sets T*_1_, …,*T_l_*, *N_c_*, *T_c_*, , *D such that:*

• *N_c_ ∪ T_c_ contains exactly the concordant mappings from M*,

• *is a valid cluster*,

*• T*_1_,…, *T_l_are valid clusters*,

• {*c*′, *T*_1_, …, *T_l_*} *and T_c_ are consistent w.r.t. two chromosome copies*, *and*

*• D is of minimal size*, *possibly empty.*

In the description above, each *T_j_* denotes a cluster of mapping assigned to a tumor chromosome, *N_c_* and *T_c_* denote subsets of concordant mappings from *M*,  denotes a set of mappings from *M* added to cluster *c*, and *D* is the set of mappings which could not be assigned to any of the four chromosomes without creating a conflict.

The method we designed to solve this problem is summarized in Algorithm 1.

We start from the overlapping components of the tumor-subset, and for each component, we proceed according to the following steps.

1) Any concordant mapping can be explained by assuming that the read pair was sequenced from the normal chromosome copy not carrying the deletion. Since no other mapping can stem from this copy, this will not influence the assignment of the discordant mappings.

2) We say that a set of mappings *supports* the normal deletion if, together with the deletion cluster from the normal data set, they call for a single deletion. We assign a maximal supporting subset of the discordant mappings to the normal chromosome. Note that, instead, we could also assign it to the respective tumor chromosome, but, in order to minimize the number of discarded mappings, it is better to leave as much space for further deletions on the tumor chromosomes as possible, as adding further mappings to a cluster refines the location and range of the deletion it indicates.

3) The remaining mappings have to be assigned to the two tumor chromosome copies, thus defining tumor-specific deletion clusters lying close to the refined normal deletion or on the other chromosome copy. In some cases, not all mappings can be embedded into a consistent assignment. We compute a partitioning with a minimal number of discarded mappings.

The result is a refined normal deletion *c*′, a set of tumor-specific deletions  and, in some cases, a set of discarded mappings*.*

Obviously, steps 1 and 2 are only stated for reasons of completeness and have not really to be carried out. Step 3 could be computed efficiently using a geometric approach [19]. Here, we relied on a simple exhaustive search. For step 5, we implemented a branch-and-bound search, which finds an optimal solution in general but only reports the best solution found so far if the search takes more than ten minutes. Experiments showed that this bounding has only a minor impact on the results.

Finally, note that, instead of building on maximal valid clusters, one could also use other models or methods, or a pre-filtered or manually curated set of normal deletions, provided that they correspond to proper deletion clusters.

## Results

The Genome Science Centre of the British Columbia Cancer Agency generated and provided paired-end mapping data from different samples taken from one adenocarcinoma patient [23]. Genome sequence data have been deposited at the European Genome-Phenome Archive (EGA) [34], accession number [EBI:EGAS00000000074].

We analyzed data from a blood sample, from now referred to as the *normal* data set, and from a skin metastasis sample, referred to as the *tumor* data set. The normal data set contained about 70-million concordant mappings (corresponding to a 4.5-fold physical coverage) and about 100-thousand discordant mappings indicating a deletion in the donor. Sequenced at higher coverage, the tumor sample contained about 160-million concordant (16.5-fold physical coverage) and about 310-thousand deletion mappings.

### Data

Sequencing was done using the Illumina Genome Analyzer II, and mapping the paired-end reads onto the human reference genome HG18 was done with the software MAQ version 0.7.1 [25].

The lengths of the fragments obtained and processed in a sequencing procedure are usually not all the same but distributed around a certain value. The distribution of the mapping lengths was used to estimate cutoffs to differentiate between *discordant* mappings and *concordant* mappings. We filtered for a minimum quality value of 20 (thresholds about 10-30 are commonly applied) and used the MAQ-preprocessor provided by the GASV software package [19] to determine the minimum (minLen) and maximum length (maxLen) for concordant mappings. Using the default options and parameters, minLen was defined as the 0.1%-quantile, and maxLen as the 99.9%-quantile. The results are summarized in Table 1.

**Table 1:** Results of the preprocessing of the mapping data (min. quality 20).

Data Set	minLen	maxLen	conc. mappings	mappings ind. a deletion
Normal	126	263	69,996,907	99,880
Tumor	90	530	158,694,096	309,134

### Inconsistency of Predicted Deletions

For the different data sets under consideration, we computed all maximal clusters, as defined in Methods section , for the given discordant mappings using GASV [19].

The normal and the tumor data were processed separately as well as pooled. In the normal data set, we found 3,928 deletion clusters. For the tumor and the pooled data set, the computation of the maximal clusters for chromosomes 1, 6, 7, 10, 18 and 19 did not finish within reasonable time due to a combinatorial explosion of the number of maximal clusters. On the remaining chromosomes, we found 46,146 (tumor) and 50,887 (pooled) deletion clusters, respectively. Deletions found in the normal data set were assumed to be patient-specific. The pooling results contained 44,276 clusters solely composed of mappings from the tumor data set, thus indicating a candidate tumor-specific deletion. We found that only 44 (1.1%) of the deletion clusters in the whole normal data set were involved in conflicts. In the tumor data set and among the candidate tumor-specific deletions in the pooled data, we found a higher level of inconsistency, as can be seen in Table 2. Note that the very large number of overlapping clusters in the disregarded chromosomes in the tumor and pooled data set comprise a high amount of conflicts, confirming this observation.

**Table 2:** Characteristics of deletion clusters and conflicts. For each considered data set as described in the text with the parameters given in Table 1, the number of maximal deletion clusters (found by GASV) and the number and percentage of clusters involved in conflicts is given. Chromosomes 1, 6, 7, 10, 18 and 19 are not included this table. (In the complete normal data set, 44 of 3,928 deletions, i.e., 1.1% were conflicting.) "Tumor-spec." denotes the set of putatively tumor-specific deletions extracted from a pooled analysis. A precise description of the notion of conflicts and the method used to detect them is given in Sections 'Haploid Conflicts' and 'Diploid Conflicts'.

Data Set	Deletions	Confl. Deletions	Del. ≥ 20	Confl. Del. ≥ 20
Normal	2,765	29 (1.0%)	1,338	23 (1.7%)
Tumor	46,146	14,234 (30.8%)	15,644	5,990 (38.3%)
Tumor-Subset	680	217 (31.9%)	628	206 (32.8%)
Pooled Tumor-spec.	44,276	13,644 (30.8%)	14,377	5,541 (38.5%)
Pooled-Subset Tumor-spec.	137	97 (70.8%)	125	92 (73.6%)

Further analysis revealed that in the tumor-subset, i.e., regions with closely located patient-specific and tumor-specific deletion clusters, the level of inconsistency was especially high: More than 70% of all putatively tumor-specific deletions were conflicting. In general, filtering for large deletions had no significant effect on the ratio of conflicting clusters. This indicates that there are inherent conflicts, not just caused by false positives candidate deletions.

To confirm these observations, we repeated the analysis on the deletion clusters obtained by BreakDancer [18]. The clusters reported by this tool are not necessarily valid according to our definition. Thus, a single cluster can be self-conflicting, i.e., a minimal conflicting set of size one. In Table 3, we report the number of self-conflicting clusters, and of those clusters contained in conflicting subsets of valid clusters. Again, we observe that there are very few conflicts in the normal data, a signal of inconsistency in the tumor data, and a higher ratio of conflicts in the tumor-subset. In general, the number of conflicts is lower compared to the analysis of the maximal valid clusters. This is because invalid clusters cannot pair with other clusters and thus increase the level of inconsistency by one. In contrast, at the same spot, several overlapping maximal valid clusters could constitute to larger conflicting sets, yielding a larger increase.

**Table 3:** Deletion clusters determined by BreakDancer and resulting conflicts. The numbers are obtained as described in Table 2. We account for two types of conflicts: Invalid (and thus self-conflicting) clusters and clusters contained in conflicting subsets of the valid clusters.

Data Set	Deletions	Confl. Deletions	Del. ≥ 20	Confl. Del. ≥ 20
Normal	949	7 + 0 (0.7%)	851	6 + 0 (0.7%)
Tumor	27,054	1,312 +102 (5.2%)	21,770	1,312 +102 (6.5%)
Tumor-Subset	476	33 + 15 (10.1%)	333	14 + 14 (8.4%)
Pooled Tumor-spec.	20,698	101 + 363 (2.2%)	16,578	50 + 250 (1.8%)
Pooled-Subset Tumor-spec.	203	1 + 12 (6.4%)	146	1 + 7 (5.5%)

### Consistent Explanation of the Data

**Figure 4:**
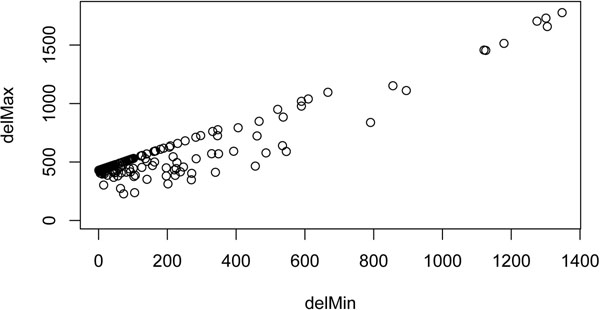
**Results: deletion ranges.** Deletion range for the 238 candidate tumor-specific deletions. Each point represents a deletion cluster: the x-coordinate is the minimum length of this deletion and the y-coordinate its maximum length. Lengths are expressed in number of nucleotides.

We applied the method described in Algorithm 1 to the tumor data. As our approach does not rely on computing maximal clusters from these data (we only need to pre-compute the maximal clusters from the normal data), all 23 chromosomes could be processed. Only 25 of the 7,579 discordant mappings in the tumor-subset had to be discarded to remove all conflicts. From the remaining mappings (99.7% of the total), a large majority (93.4%) supported and refined 928 normal deletions. The remaining 526 mappings defined 238 candidate tumor-specific deletions. As can be seen in Figure 4, the range of deletion length for these 238 deletion clusters lies in the order of a few hundred bases.

**Figure 5:**
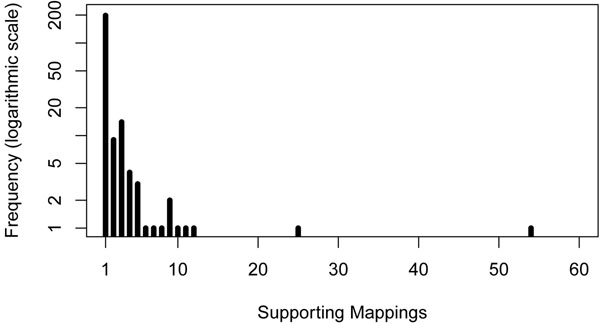
**Results: Support. Support for the 238 candidate tumor-specific deletions.** The support is defined by the number of mappings in the cluster defining each deletion.

In Figure 5, we show a histogram of the sizes of the cluster (number of mappings) supporting these candidate tumor-specific deletions. We can notice a large number of small size clusters. It is however important to note that a tumor-specific deletion might be supported by further mappings which have been assigned to the normal deletion by our greedy approach. That is why we refrained from filtering the obtained tumor-specific clusters for a minimal size. This raises the interesting problem to refine and increase the support for potential tumor-specific deletions by re-assigning to the tumor chromosomes mappings assigned to the normal chromosome.

**Figure 6:**
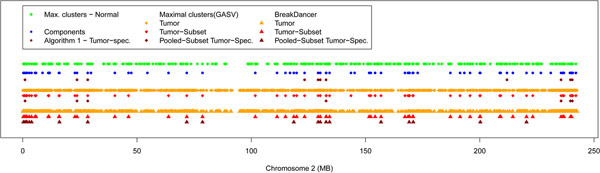
**Results: Comparison with GASV and BreakDancer.** Deletion clusters inferred by Algorithm 1, GASV and BreakDancer on chromosome 2. Note that many deletions in close proximity may appear as a single dot, and the size of a dot is in general larger than the respective deletion. Illustrations for all chromosomes can be found in Additional file 2.

Figure 6 illustrates the result of our method, GAS V and BreakDancer on chromosome 2. We can notice that Algorithm 1 agrees when both GASV and BreakDancer call for a potential tumor-specific deletion, but detects additional candidates that are supported by one or none of the two other methods. Generally, Algorithm 1 agrees more with GASV, which can be explained by the fact that they both rely on the notion of valid clusters, unlike BreakDancer. More precisely, any potential tumor-specific deletion found by analyzing the pooled data set with GASV is also called by our approach. Indeed, for a given overlapping component, if all mappings from the tumor sample can be consistently added to the normal deletion by Algorithm 1, then there is exactly one maximal valid cluster found by GASV that contains all tumor and all normal mappings, and is thus not considered as a tumor-specific deletion. Illustrations for all chromosomes can be found in Additional file 2.

**Figure 7:**
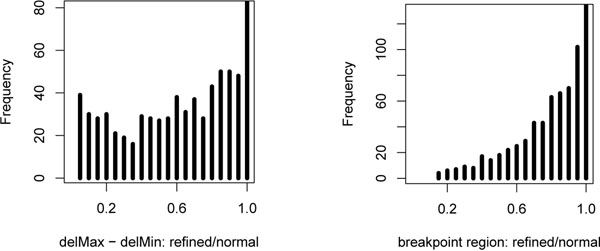
**Results: refinement of deletion and breakpoint region sizes.** (Left) Ratio between the initial deletion size and the deletion size after processing, for clusters indicating normal deletions. (Right) Ratio between the initial breakpoint region length and the breakpoint region length after processing, for clusters indicating normal deletions.

We also looked at the refined normal deletions, i.e., the clusters of mappings from both the normal and tumor data sets that agreed with a normal deletion cluster. Figure 7 below shows how the deletion length and breakpoint region were modified by including possible mappings from the mixed data into the normal clusters. We can observe a significant refinement of the characteristics of normal deletions.

For 2,896 (75.7%) normal deletions, we did not find any supporting mapping in the tumor data set. Further investigations revealed that almost all of these were either very small deletions (minimal deletion size < 50), or were defined by only two mappings in the normal data set, or overlapped segmental duplicated regions of a chromosome. This suggests that they might be false positives or that the region in the tumor data set was not sufficiently covered (<2 overlapping concordant mappings).

**Figure 8:**
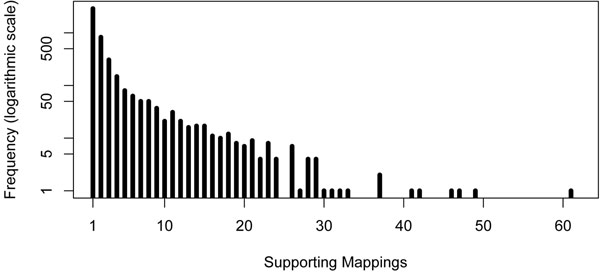
**Results: Singletons.** aSupported normal singletons. In this histogram, the number of normal deletions defined by exactly one mapping is shown w.r.t. the number of mappings in the tumor data set supporting the deletion. E.g., there were 50 singletons supported by eight mappings. The numbers are based on the tumor-subset only. Chromosome 18 is not included, because the computation did not finish within reasonable time limits.

However, a closer look at the many normal deletions which were defined by only a small number of mappings from the normal data set showed that many of them are supported by mappings from the tumor data set. For example, Figure 8 shows that a significant number of normal singletons (clusters of size one) are well supported by mappings from the tumor data set (that was sequenced with a much higher coverage). This indicates that many singletons might be true positive rather than false positive normal deletions.

To some extent, this can be a result of the roughly four times higher coverage of the tumor sample. Another possible explanation for the observation of many singletons with a significantly high support by the tumor data might be a contaminated normal sample: If the normal sample contained only very few tumor cells, then, in the sequencing process, some tumor-specific deletions might have been sequenced with very low coverage, resulting in only a few or even single discordant read pairs calling these deletions. In contrast, the cancer sample contained many more tumor cells, yielding a high coverage and thus high support for the same deletions. Recall that the tumor sample was actually taken from a skin metastasis. Since cancer spreads via the lymphatic system or the bloodstream, this also supports the hypothesis. In all cases, this points to candidate tumor-specific deletions and specific cases that might require improved detection methods. So here again, the question of assigning the mappings from a contaminated (by non-tumor cells) tumor sample between the normal and tumor chromosomes copies comes naturally to mind.

## Conclusion

We analyzed two data sets obtained from different tissues of the same patient: A tumor and a normal sample. Paired-end reads were mapped to a reference genome and discordant mappings were assembled to maximal valid clusters calling for deletions (using GASV [19]). These deletions were then analyzed in terms of consistency: Can all mappings and deletions be explained by assuming that all read pairs were read from the mapped positions in a diploid set of chromosomes? We described a simple combinatorial model of deletion consistency that refines the previous work of [24]. We found that, in this model, the deletions predicted for the normal data are almost consistent. In contrast, the tumor data set showed a higher level of inconsistency. For those regions of the genome harboring a normal deletion, we found the highest rate of conflicting deletions among putatively tumor-specific deletions. We thus focused our study on this *tumor-subset.*

Usually a tumor sample also contains normal cells. Thus, instead of a diploid set of chromosomes, we expect four copies of each chromosome (diploid normal and diploid tumor genome). Based on this assumption, and our consistency model, we described the problem of consistent explanation of the data as an optimization problem and described a method to solve it. Applying this method on our data, we were able to explain almost all the considered mapping data in a consistent way. Only very few mappings could not be explained (0.3%), most of the mappings supported inherent normal deletions (92.7%), and 6.9% were clustered to 238 tumor-specific deletions.

Summarized, we revealed that deletion calls obtained by standard methods possess a high rate of inconsistency, and we present a model that, even though built on strict assumptions, such as diploidy of the genomes and each deletion cluster spanning exactly one deletion, allows for an almost consistent explanation of the data.

Although these results look promising, they raise some questions. Possible reasons for false positive deletion calls and thus inconsistencies are misleading pairs of reads, for instance caused by chimeric fragments in the sequencing procedure, or erroneous mappings, for instance caused by repeated regions in the genome. Even though we filtered for unambiguous mappings of high quality, some wrong mappings were probably left. Introducing read error-correction in the general process of mapping assignment is a natural extension. Next, it is not unlikely to find polyploid chromosomes with three or more copies in tumor cells. Furthermore, the sample could contain cells from different tumor lineages—also indicating a higher copy number. Certainly, assuming further chromosome copies, all mappings could eventually be assigned consistently. However, including copy number information to refine the assumptions would be a first way to bring our model closer to reality. And, as already mentioned, our model does not include loss of heterozygosity (LOH) so far. Regions which remain in conflict might also be putative LOH sites.

Another approach to explain more mappings is to relax the definition of consistency. In particular, the assumption that each cluster only spans exactly one deletion is mainly of technical origin and arguable. If we would allow any deletion being split into any number of smaller fragments, the problem complexity would increase. However, we believe the problem might be somewhat tractable for an intermediate model where at most *k* splits are allowed.

One issue which is not addressed by our study so far is the discrimination of heterozygous and homozygous deletions. A deletion affecting only one copy of a chromosome allows further deletions on the second copy. In contrast, a homozygous deletion residing on both copies, might also occur on both copies of the tumor chromosomes, leaving only limited space for further, tumor-specific deletions. This creates a higher potential for conflicts. Hence, any mis-classification as homozygous could falsely increase the number of discarded mappings dramatically. We currently investigate how homozygosity can be securely included into our method. A possible approach would be to first consider normal deletions as heterozygous, as we do in the present work, then to analyze each configuration produced by our method (a set of mappings defining a refined normal deletion and possible closely located tumor-specific deletions) and to investigate the homozygous/heterozygous issue in a post-processing phase. Such a post-processing could also address the issue of re-assigning mappings from the normal chromosomes to the tumor chromosomes that we raised in discussing our results. More generally, our approach is more intended to provide a first level of conflict clearing and to highlight potential region where tumor-specific deletions could have occurred close to a normal deletion. Post-processing the results of our method to refine them, without re-creating conflicts, would be a natural extension.

We also plan to extend our framework to cover overlapping normal deletions and other more complicated cases which we discarded so far. Although these only constitute a minority (2.9% of the normal deletions), their complex structure might comprise a lot of conflicts and is thus worth to study.

Next, the combinatorics of mappings, clusters and conflicts is still very poorly understood and require further investigation, in particular to find properties that will lead to efficient algorithms to detect conflicts. Also, several combinatorial optimization problems we considered in the present work still await for efficient algorithms (formally stated in Appendix A).

In general, paired-end mapping approaches can be used to analyze different types structural variations. Deletions certainly belong to the simplest classes, well suitable as a starting point to study structural variation, but far from representing the whole spectrum of cancer development. As a next step, we intend to include further types of variations to approach a more complete model, which will however require combinatorial developments.

## List of abbreviations

LOH: Loss of Heterozygosity.

## Competing interests

The authors declare that they have no competing interests.

## Authors' contributions

R.W. and C.C. designed the method. R.W. implemented the method. R.W. and C.C. wrote the manuscript. All authors read and approved the final manuscript.

## Appendix A: Statements of algorithmic and combinatorial optimization problems

Problem 1: Detection of minimal conflicting sets.

**Given:** Set of mappings *M*, set of valid clusters *C.*

**Task:** Find all minimal conflicting subsets of *M* ∪ *C* w.r.t. a diploid genome.

**Remark:** General, but exponential time, techniques were described in [26,27], for the different problem of reconstructing ancestral genomes and gene clusters, but they can be applied for deletion clusters. As far as we know, ad-hoc approaches for the specific case of deletion clusters have not been investigated.

Problem 2: Test for consistency of clusters in the haploid case allowing for splitting each given deletion into *k* smaller deletions.

**Given:** Set of clusters *C.*

**Task:** Find (if existing) a set *D* of positions such that for each cluster *c* ∈ *C:*

*• delMin*(*c*) ≤ |*D* ∩ *br*(*c*)| ≤ *delMax*(*c*), and

• *D* ∩ *br*(*c*) is a set of at most *k* intervals.

**Remark:** The set *D* represents the genomic positions deleted in the donor genome. Strictly speaking, positions onto which any read maps have to be excluded from *D.* To simplify the verification one can simply discard all these position in general and adjust *delMin*(*c*), *delMax*(*c*) and *br*(*c*) for each cluster *c* correspondingly.

Problem 3: Finding a maximum set of concordant mappings for a deletion cluster

**Given:** Set of discordant mappings *M_d_*, valid deletion cluster *c*.

**Task:** Find a maximum subset  of *M_d_* such that  is a valid cluster.

**Remark:** The geometric framework described in [19] allows to solve this problem.

Problem 4: Consistent assignment to four chromosome copies.

**Given:** Set of mappings *M*, valid cluster *c*.

**Task:** Find a partition of *M* into disjoint sets , *D* such that:

•  are valid clusters,

•  is consistent w.r.t. one chromosome copy, and

•  is consistent w.r.t. one chromosome copy, and

• *D* is of minimal size, possibly empty.

## Supplementary Material

Additional file 1 Implementation of the method to find a consistent explanation for a (mixed) tumor-subset under consideration of associated normal data (Algorithm 1). This ZIP-archive contains the main algorithm and a converter as Java Archives (JARs), and a text file with detailed instructions. Click here for file

Additional file 2 Illustration of deletion clusters inferred by Algorithm 1, GASV and BreakDancer. Note that many deletions in close proximity may appear as a single dot, and the size of a dot is in general larger than the respective deletion. For some data sets, the computation of all maximal clusters was infeasible. This ZIP-archive contains a PDF file for each chromosome. Click here for file
