# Supplementary figures and images for: Consistency-based detection of potential tumor-specific deletions in matched normal/tumor genomes
Source: BMC Bioinformatics. 2011 Oct 5;12(Suppl 9):S21. doi: 10.1186/1471-2105-12-S9-S21 (PMC3283309; doi:10.1186/1471-2105-12-S9-S21)

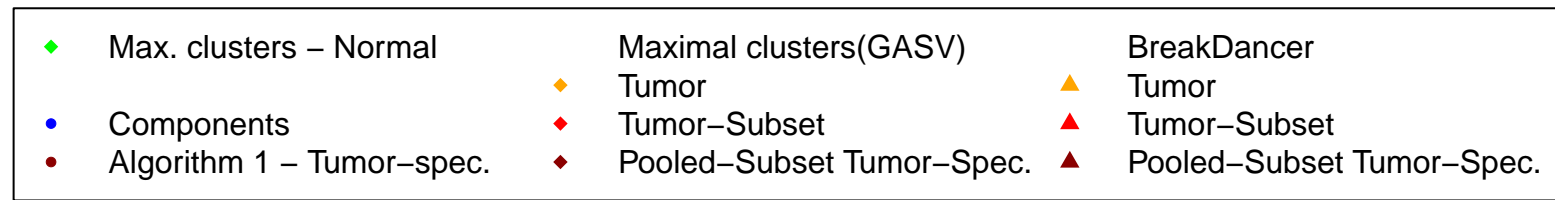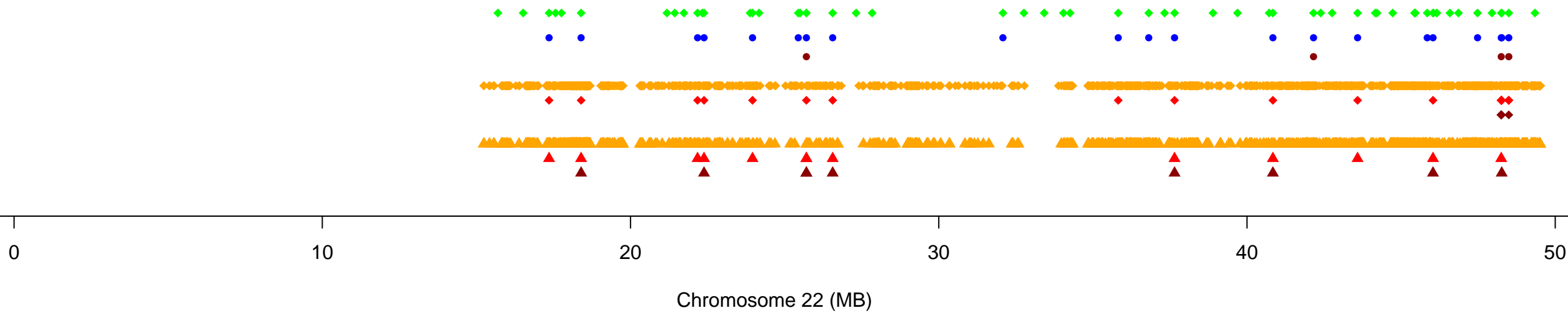

Supplement: Additional file 2 — Illustration of deletion clusters inferred by Algorithm 1, GASV and BreakDancer. Note that many deletions in close proximity may appear as a single dot, and the size of a dot is in general larger than the respective deletion. For some data sets, the computation of all maximal clusters was infeasible. This ZIP-archive contains a PDF file for each chromosome. [file 1471-2105-12-S9-S21-S2.zip › allChromosomesWithLegend/chr22WithLegend.pdf]

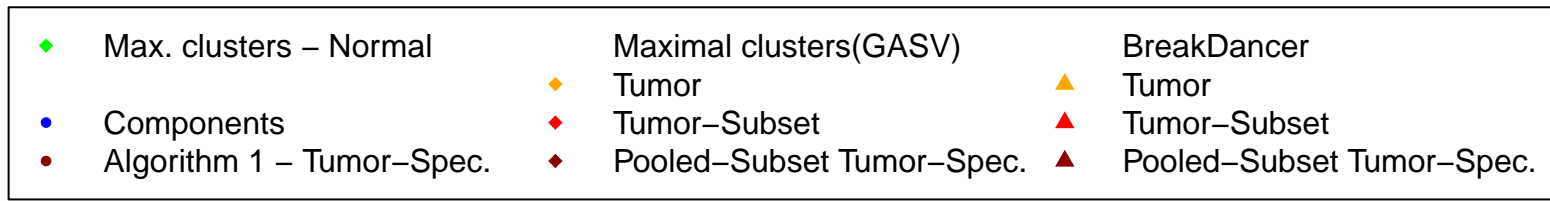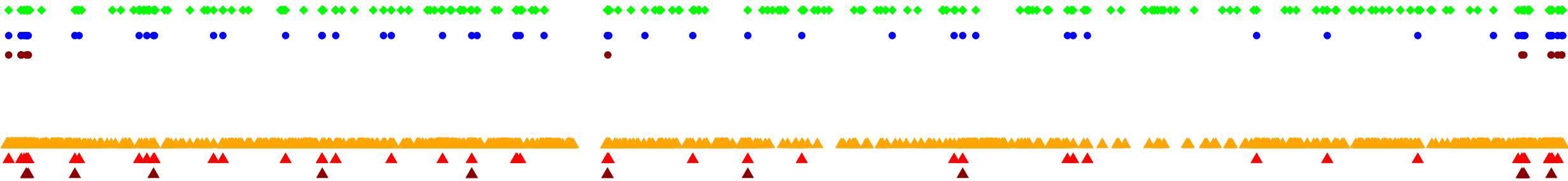

Chromosome 7 (MB)

Supplement: Additional file 2 — Illustration of deletion clusters inferred by Algorithm 1, GASV and BreakDancer. Note that many deletions in close proximity may appear as a single dot, and the size of a dot is in general larger than the respective deletion. For some data sets, the computation of all maximal clusters was infeasible. This ZIP-archive contains a PDF file for each chromosome. [file 1471-2105-12-S9-S21-S2.zip › allChromosomesWithLegend/chr7WithLegend.pdf]

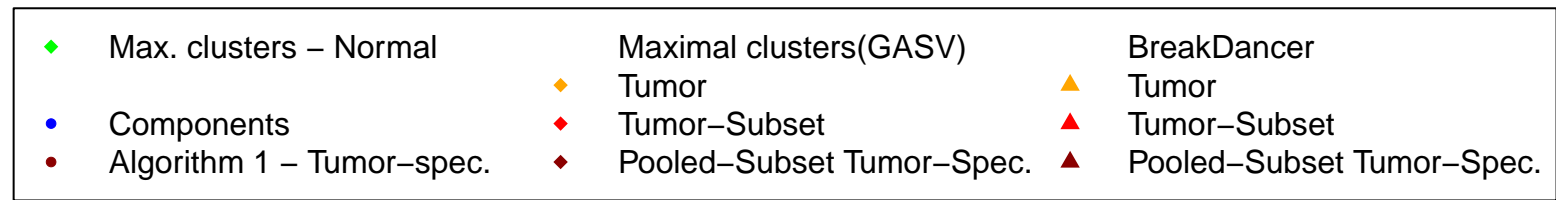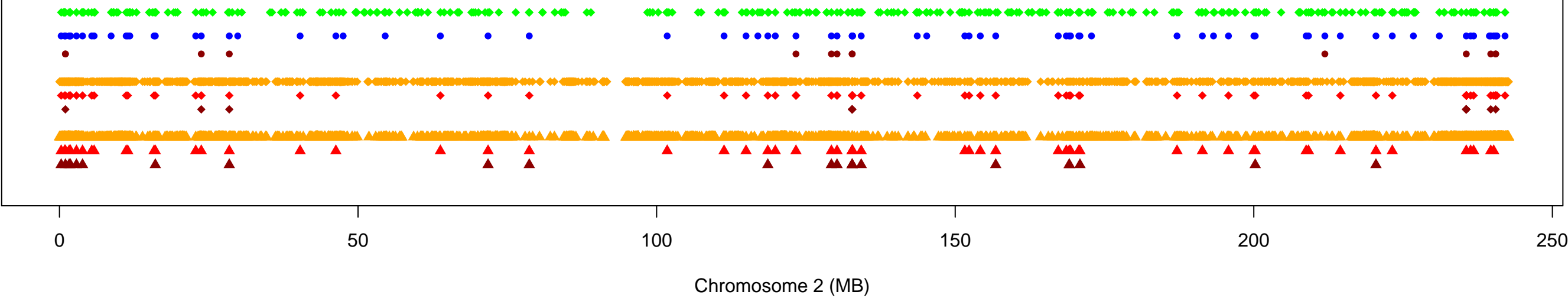

Supplement: Additional file 2 — Illustration of deletion clusters inferred by Algorithm 1, GASV and BreakDancer. Note that many deletions in close proximity may appear as a single dot, and the size of a dot is in general larger than the respective deletion. For some data sets, the computation of all maximal clusters was infeasible. This ZIP-archive contains a PDF file for each chromosome. [file 1471-2105-12-S9-S21-S2.zip › allChromosomesWithLegend/chr2WithLegend.pdf]

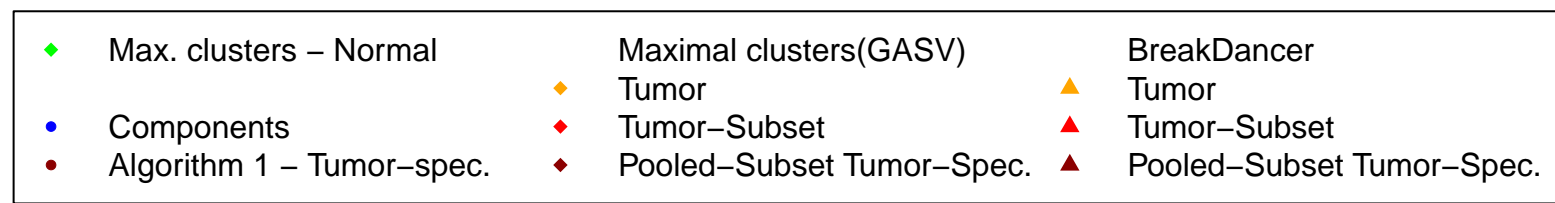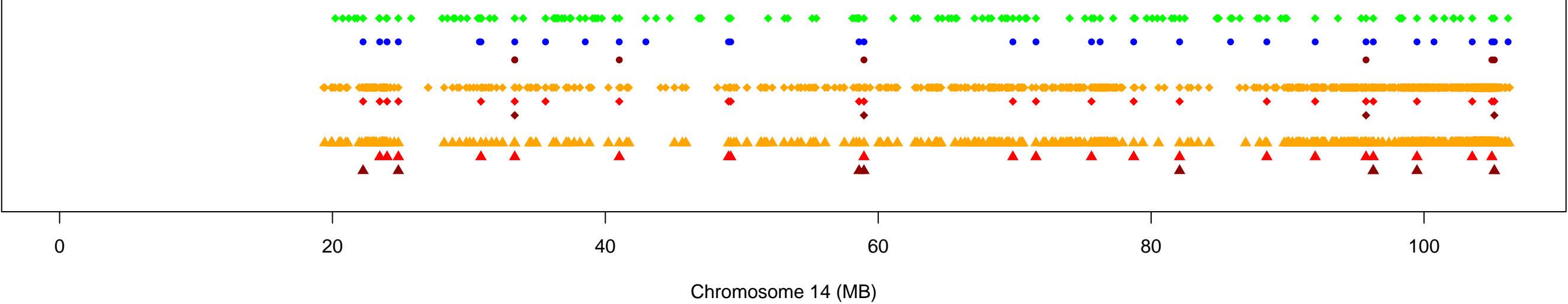

Supplement: Additional file 2 — Illustration of deletion clusters inferred by Algorithm 1, GASV and BreakDancer. Note that many deletions in close proximity may appear as a single dot, and the size of a dot is in general larger than the respective deletion. For some data sets, the computation of all maximal clusters was infeasible. This ZIP-archive contains a PDF file for each chromosome. [file 1471-2105-12-S9-S21-S2.zip › allChromosomesWithLegend/chr14WithLegend.pdf]

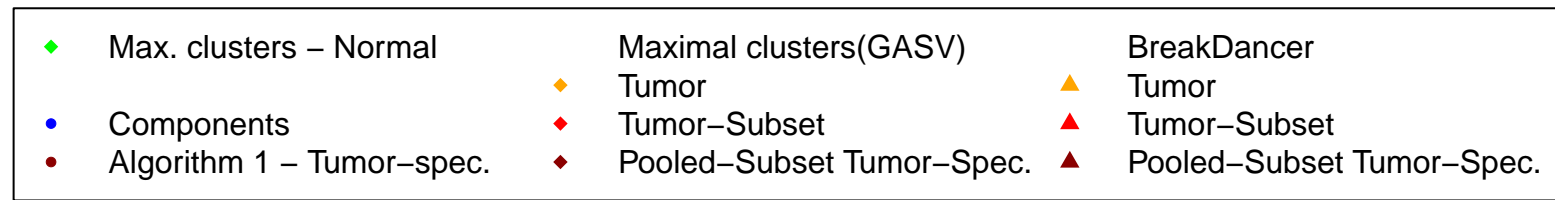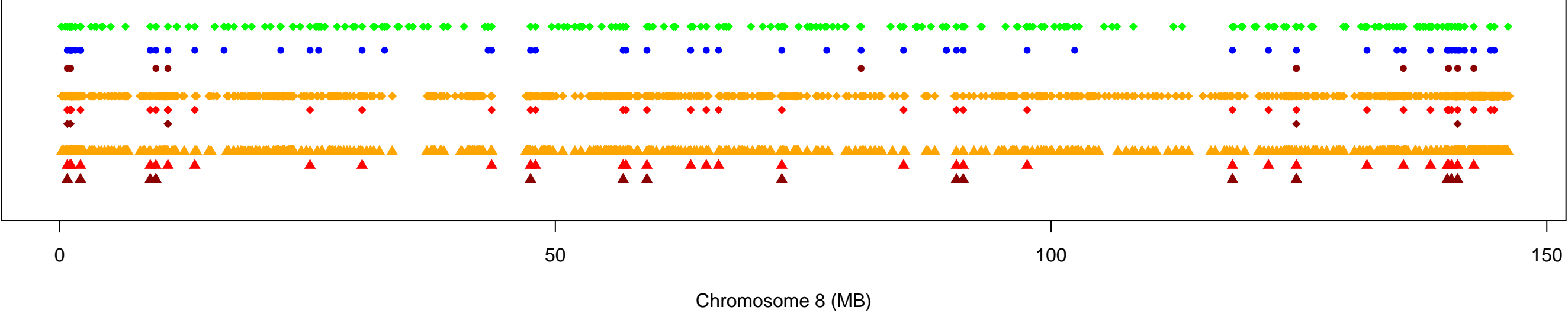

Supplement: Additional file 2 — Illustration of deletion clusters inferred by Algorithm 1, GASV and BreakDancer. Note that many deletions in close proximity may appear as a single dot, and the size of a dot is in general larger than the respective deletion. For some data sets, the computation of all maximal clusters was infeasible. This ZIP-archive contains a PDF file for each chromosome. [file 1471-2105-12-S9-S21-S2.zip › allChromosomesWithLegend/chr8WithLegend.pdf]

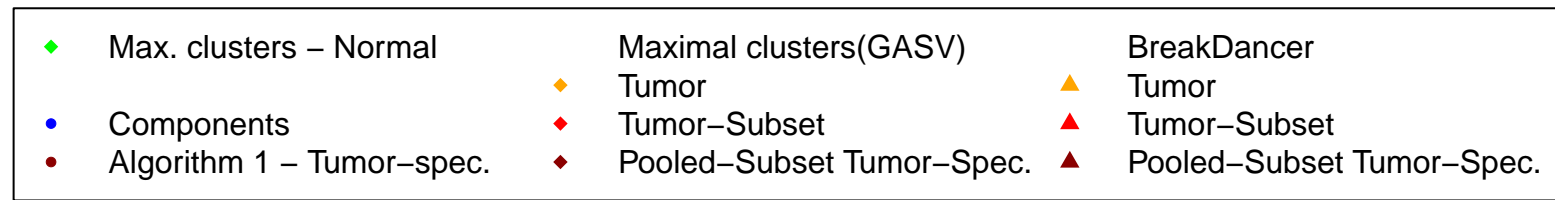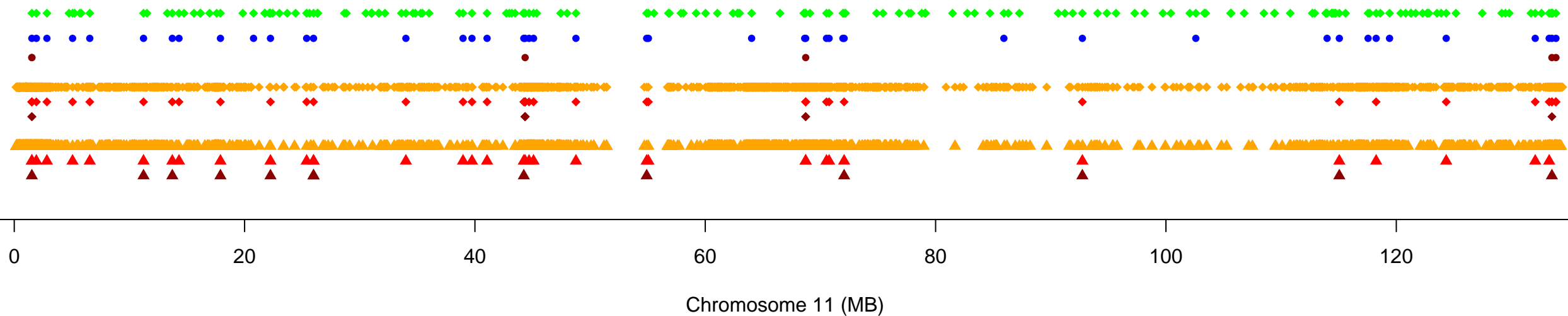

Supplement: Additional file 2 — Illustration of deletion clusters inferred by Algorithm 1, GASV and BreakDancer. Note that many deletions in close proximity may appear as a single dot, and the size of a dot is in general larger than the respective deletion. For some data sets, the computation of all maximal clusters was infeasible. This ZIP-archive contains a PDF file for each chromosome. [file 1471-2105-12-S9-S21-S2.zip › allChromosomesWithLegend/chr11WithLegend.pdf]

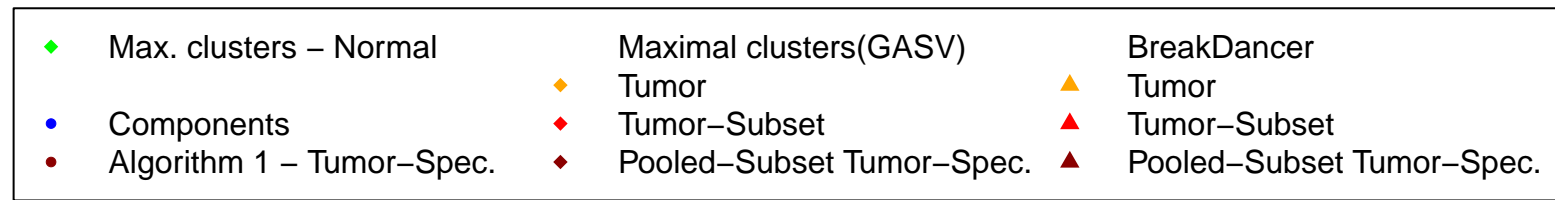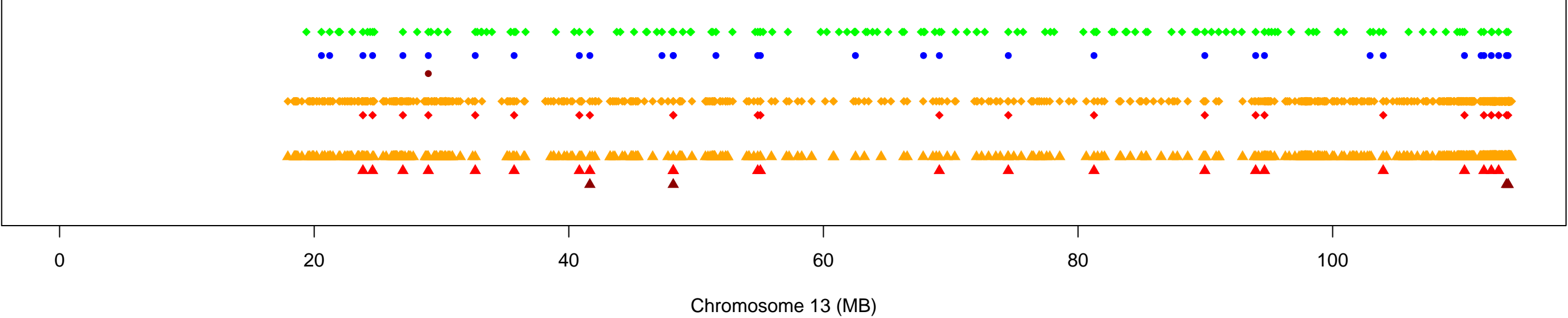

Supplement: Additional file 2 — Illustration of deletion clusters inferred by Algorithm 1, GASV and BreakDancer. Note that many deletions in close proximity may appear as a single dot, and the size of a dot is in general larger than the respective deletion. For some data sets, the computation of all maximal clusters was infeasible. This ZIP-archive contains a PDF file for each chromosome. [file 1471-2105-12-S9-S21-S2.zip › allChromosomesWithLegend/chr13WithLegend.pdf]

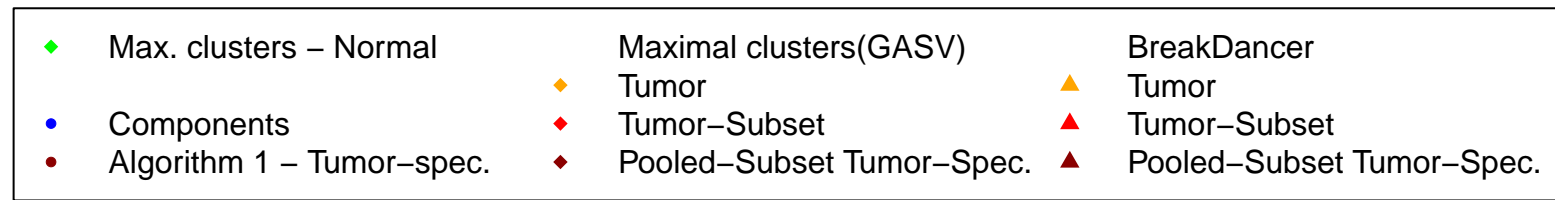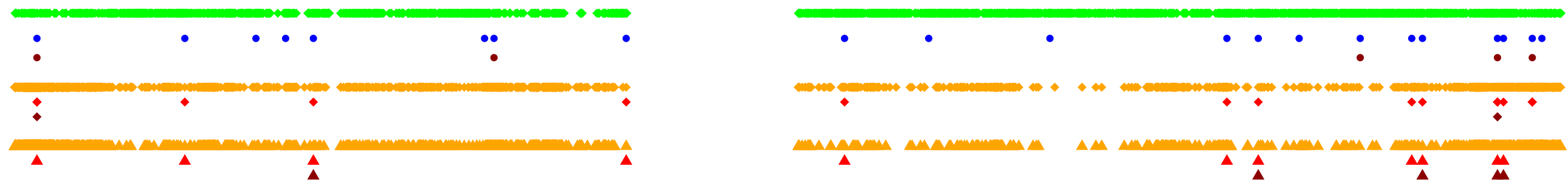

0

20

40

60

80

Chromosome 16 (MB)

Supplement: Additional file 2 — Illustration of deletion clusters inferred by Algorithm 1, GASV and BreakDancer. Note that many deletions in close proximity may appear as a single dot, and the size of a dot is in general larger than the respective deletion. For some data sets, the computation of all maximal clusters was infeasible. This ZIP-archive contains a PDF file for each chromosome. [file 1471-2105-12-S9-S21-S2.zip › allChromosomesWithLegend/chr16WithLegend.pdf]

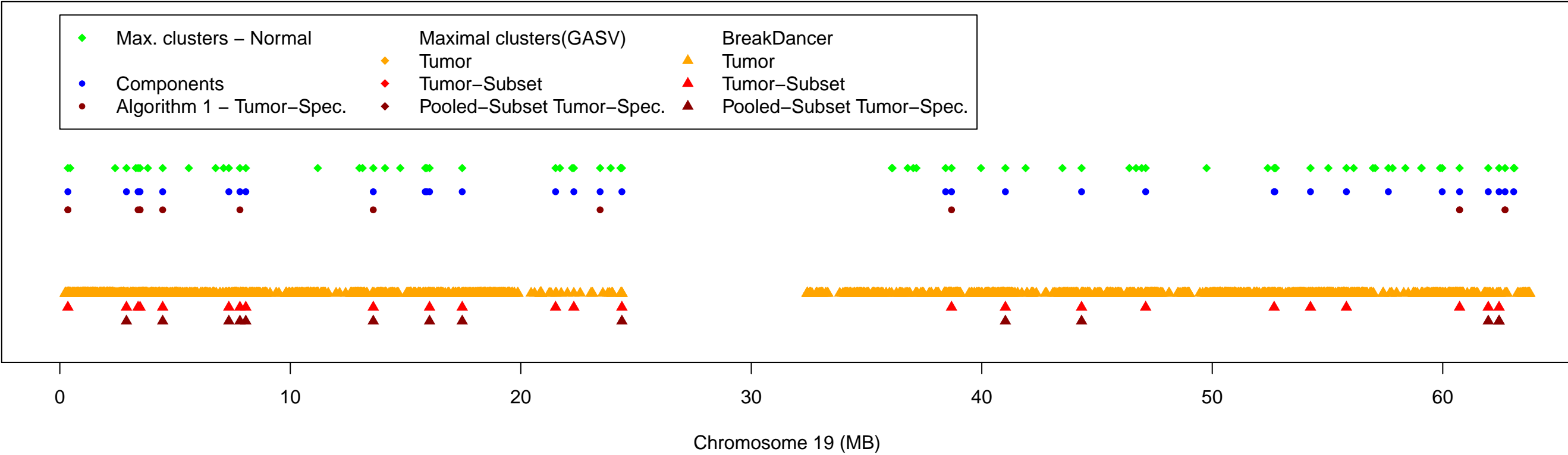

Supplement: Additional file 2 — Illustration of deletion clusters inferred by Algorithm 1, GASV and BreakDancer. Note that many deletions in close proximity may appear as a single dot, and the size of a dot is in general larger than the respective deletion. For some data sets, the computation of all maximal clusters was infeasible. This ZIP-archive contains a PDF file for each chromosome. [file 1471-2105-12-S9-S21-S2.zip › allChromosomesWithLegend/chr19WithLegend.pdf]

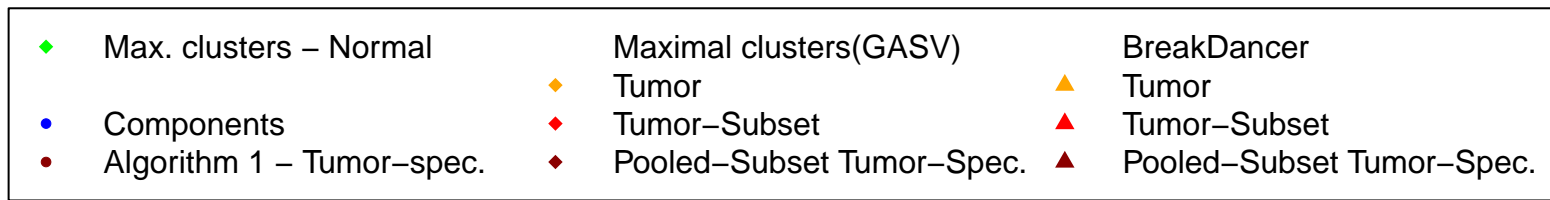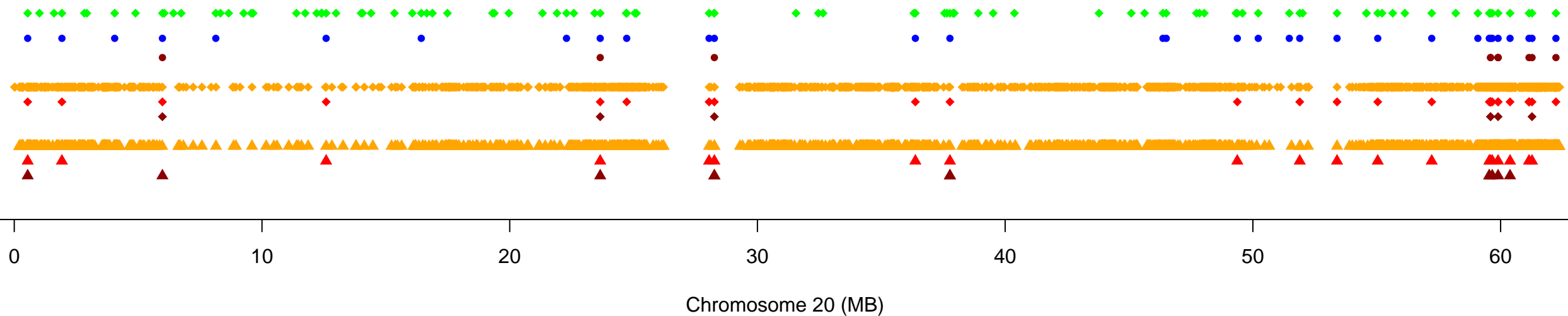

Supplement: Additional file 2 — Illustration of deletion clusters inferred by Algorithm 1, GASV and BreakDancer. Note that many deletions in close proximity may appear as a single dot, and the size of a dot is in general larger than the respective deletion. For some data sets, the computation of all maximal clusters was infeasible. This ZIP-archive contains a PDF file for each chromosome. [file 1471-2105-12-S9-S21-S2.zip › allChromosomesWithLegend/chr20WithLegend.pdf]

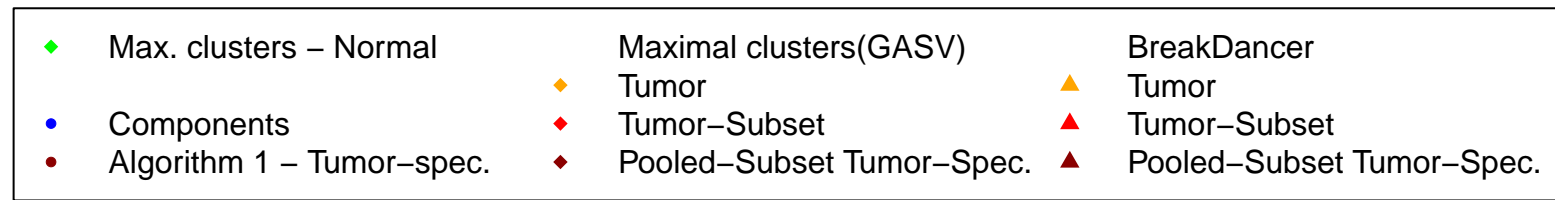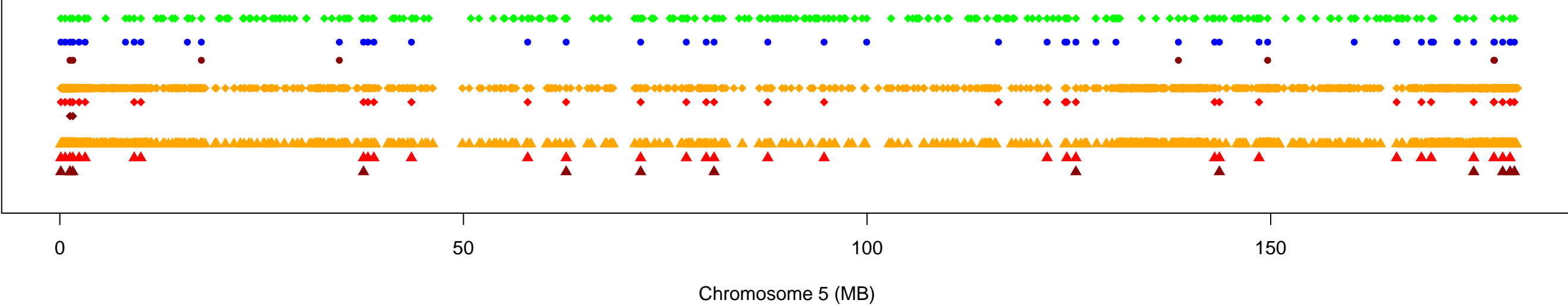

Supplement: Additional file 2 — Illustration of deletion clusters inferred by Algorithm 1, GASV and BreakDancer. Note that many deletions in close proximity may appear as a single dot, and the size of a dot is in general larger than the respective deletion. For some data sets, the computation of all maximal clusters was infeasible. This ZIP-archive contains a PDF file for each chromosome. [file 1471-2105-12-S9-S21-S2.zip › allChromosomesWithLegend/chr5WithLegend.pdf]

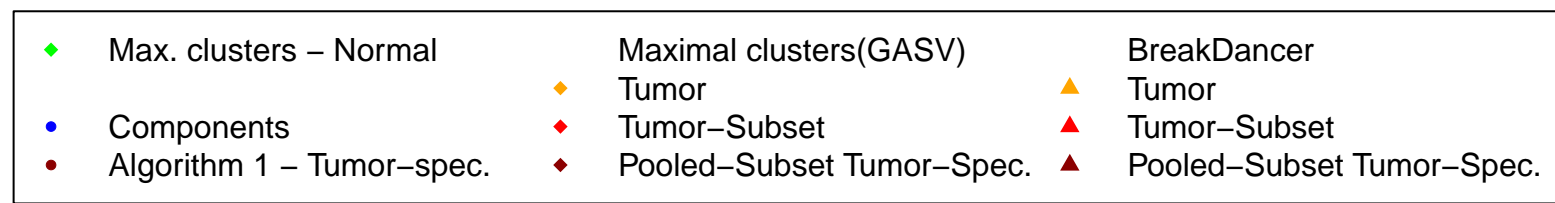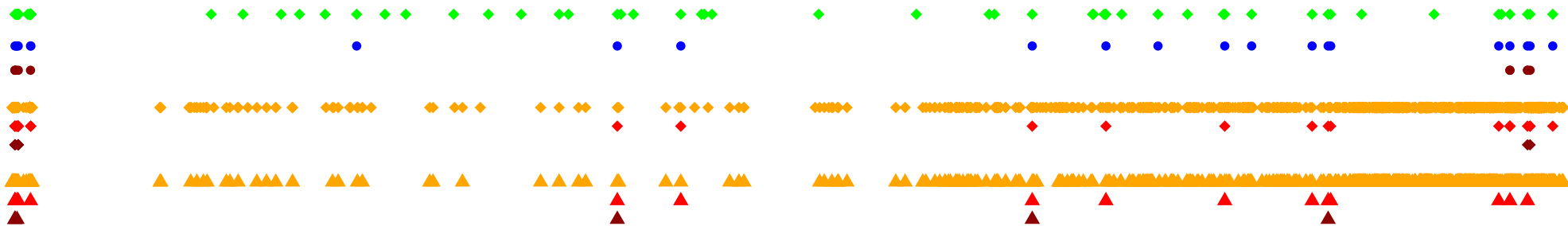

0

10

20

30

40

Chromosome 21 (MB)

Supplement: Additional file 2 — Illustration of deletion clusters inferred by Algorithm 1, GASV and BreakDancer. Note that many deletions in close proximity may appear as a single dot, and the size of a dot is in general larger than the respective deletion. For some data sets, the computation of all maximal clusters was infeasible. This ZIP-archive contains a PDF file for each chromosome. [file 1471-2105-12-S9-S21-S2.zip › allChromosomesWithLegend/chr21WithLegend.pdf]

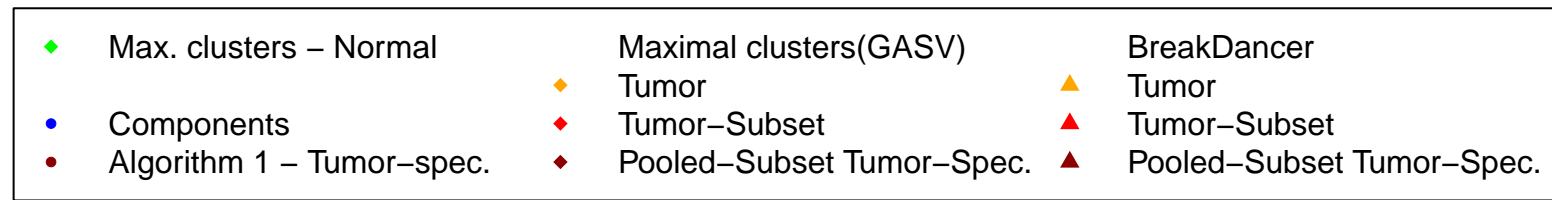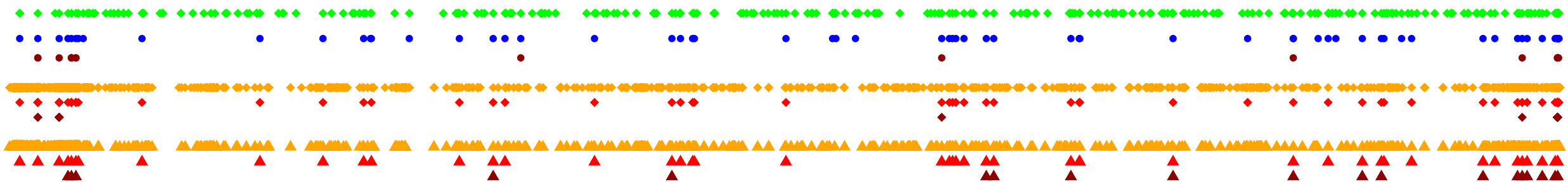

0

50

100

150

Chromosome 4 (MB)

Supplement: Additional file 2 — Illustration of deletion clusters inferred by Algorithm 1, GASV and BreakDancer. Note that many deletions in close proximity may appear as a single dot, and the size of a dot is in general larger than the respective deletion. For some data sets, the computation of all maximal clusters was infeasible. This ZIP-archive contains a PDF file for each chromosome. [file 1471-2105-12-S9-S21-S2.zip › allChromosomesWithLegend/chr4WithLegend.pdf]

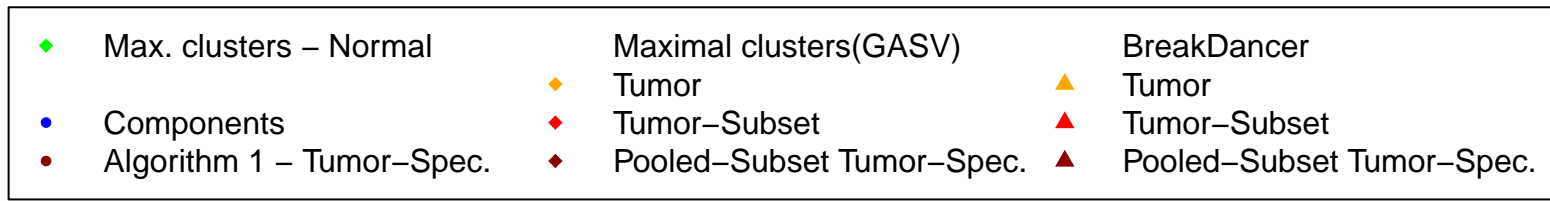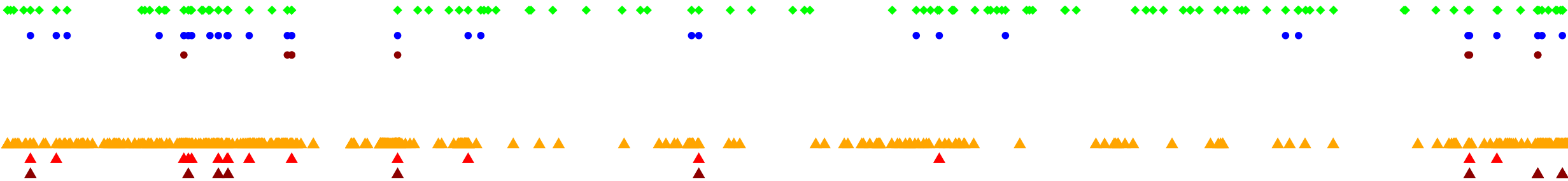

0 20 40 60

Chromosome 18 (MB)

Supplement: Additional file 2 — Illustration of deletion clusters inferred by Algorithm 1, GASV and BreakDancer. Note that many deletions in close proximity may appear as a single dot, and the size of a dot is in general larger than the respective deletion. For some data sets, the computation of all maximal clusters was infeasible. This ZIP-archive contains a PDF file for each chromosome. [file 1471-2105-12-S9-S21-S2.zip › allChromosomesWithLegend/chr18WithLegend.pdf]

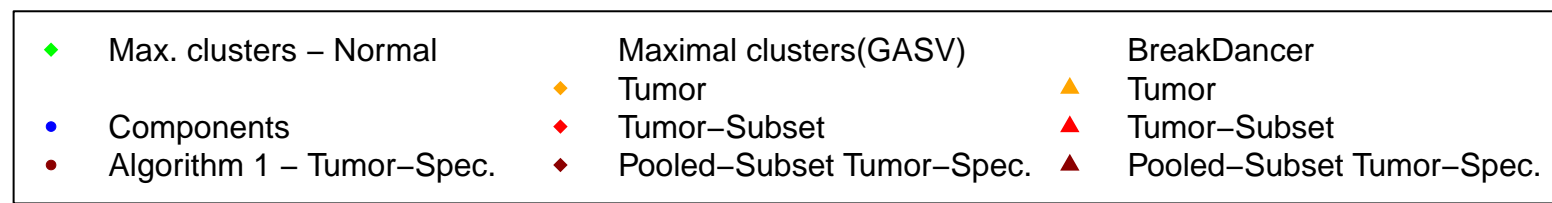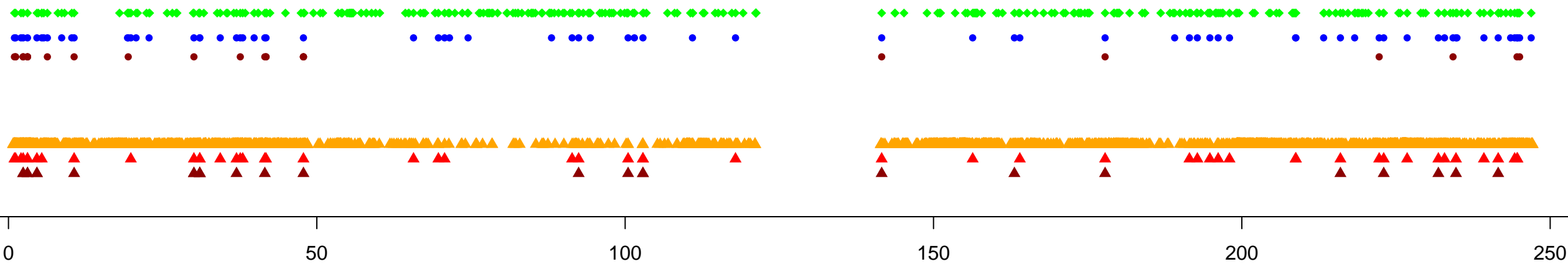

Supplement: Additional file 2 — Illustration of deletion clusters inferred by Algorithm 1, GASV and BreakDancer. Note that many deletions in close proximity may appear as a single dot, and the size of a dot is in general larger than the respective deletion. For some data sets, the computation of all maximal clusters was infeasible. This ZIP-archive contains a PDF file for each chromosome. [file 1471-2105-12-S9-S21-S2.zip › allChromosomesWithLegend/chr1WithLegend.pdf]

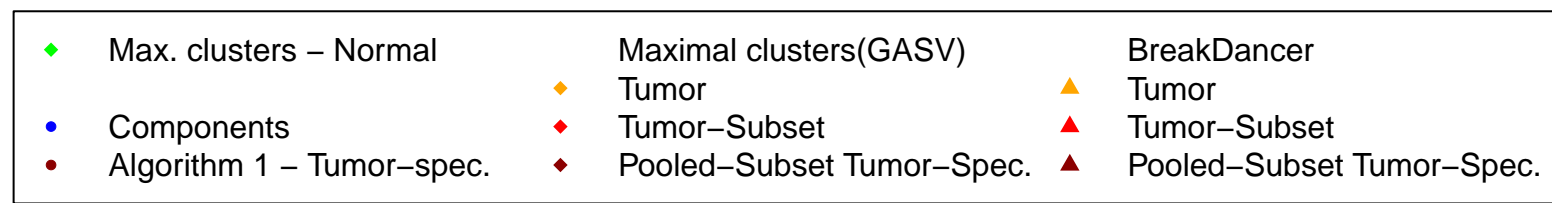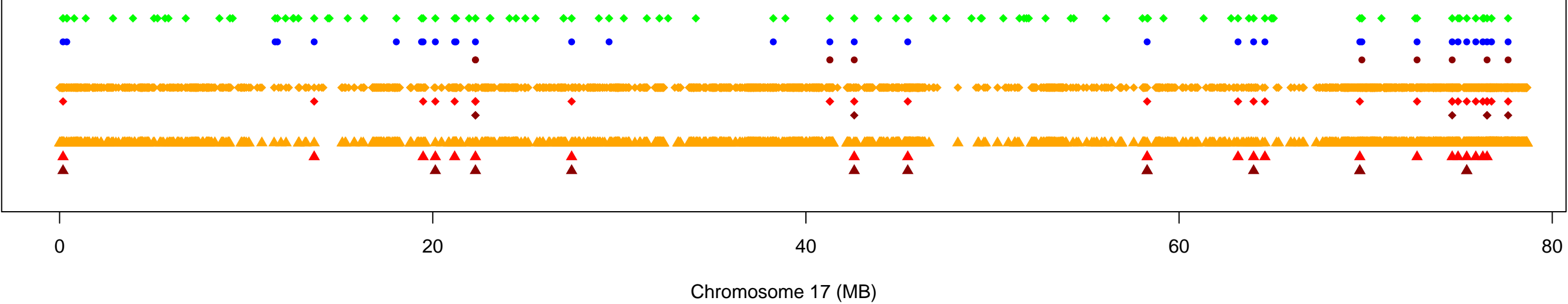

Supplement: Additional file 2 — Illustration of deletion clusters inferred by Algorithm 1, GASV and BreakDancer. Note that many deletions in close proximity may appear as a single dot, and the size of a dot is in general larger than the respective deletion. For some data sets, the computation of all maximal clusters was infeasible. This ZIP-archive contains a PDF file for each chromosome. [file 1471-2105-12-S9-S21-S2.zip › allChromosomesWithLegend/chr17WithLegend.pdf]

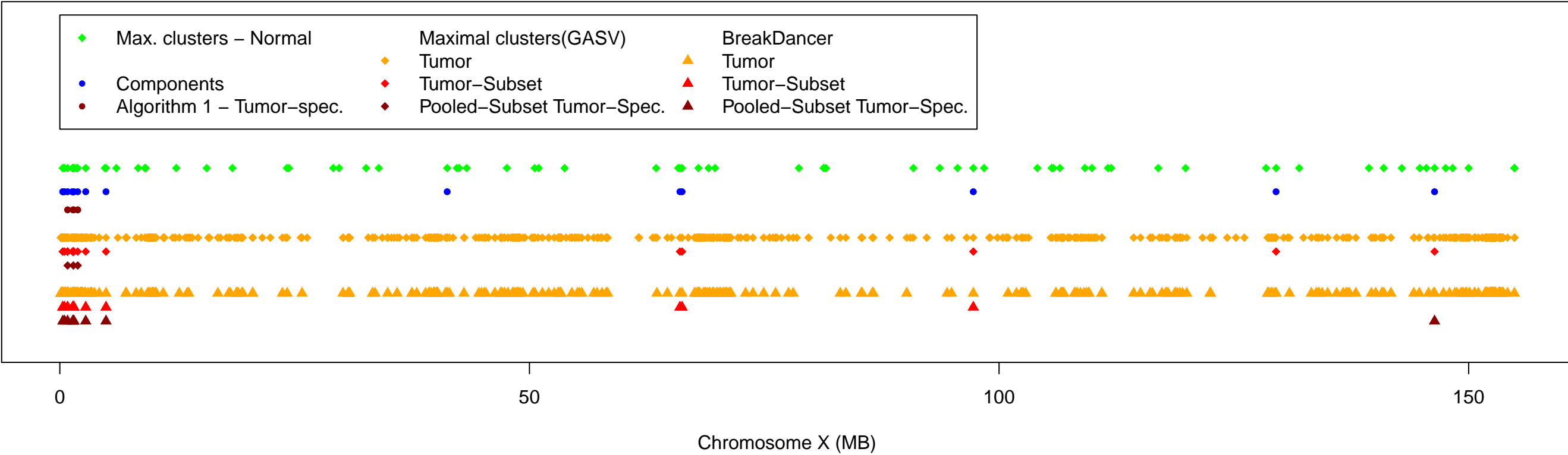

Supplement: Additional file 2 — Illustration of deletion clusters inferred by Algorithm 1, GASV and BreakDancer. Note that many deletions in close proximity may appear as a single dot, and the size of a dot is in general larger than the respective deletion. For some data sets, the computation of all maximal clusters was infeasible. This ZIP-archive contains a PDF file for each chromosome. [file 1471-2105-12-S9-S21-S2.zip › allChromosomesWithLegend/chrXWithLegend.pdf]

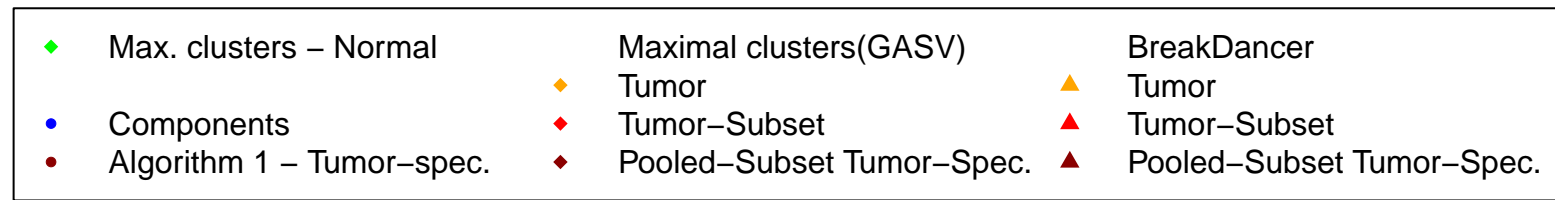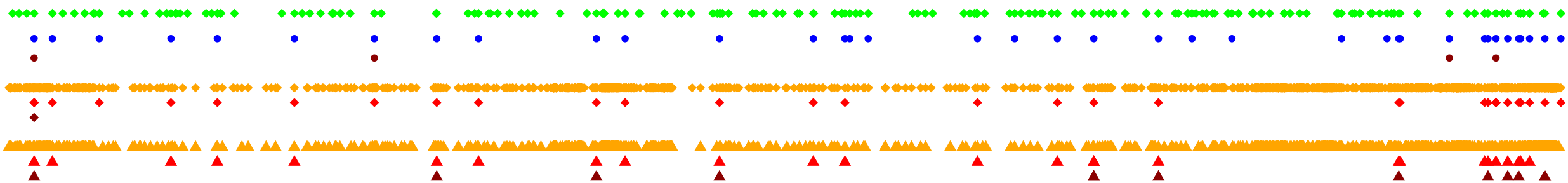

Chromosome 12 (MB)

Supplement: Additional file 2 — Illustration of deletion clusters inferred by Algorithm 1, GASV and BreakDancer. Note that many deletions in close proximity may appear as a single dot, and the size of a dot is in general larger than the respective deletion. For some data sets, the computation of all maximal clusters was infeasible. This ZIP-archive contains a PDF file for each chromosome. [file 1471-2105-12-S9-S21-S2.zip › allChromosomesWithLegend/chr12WithLegend.pdf]

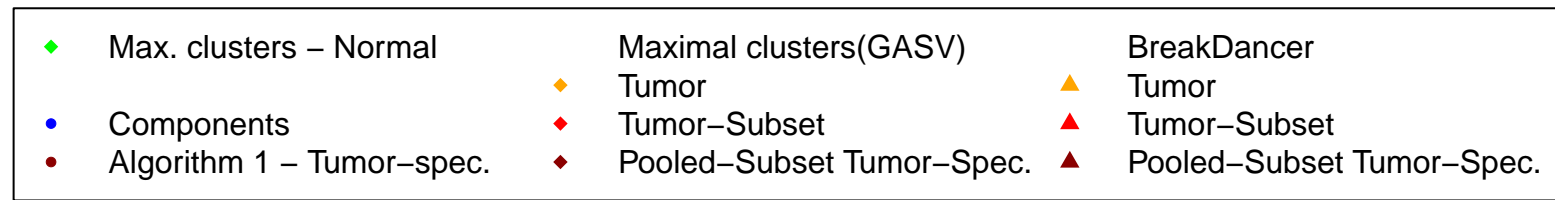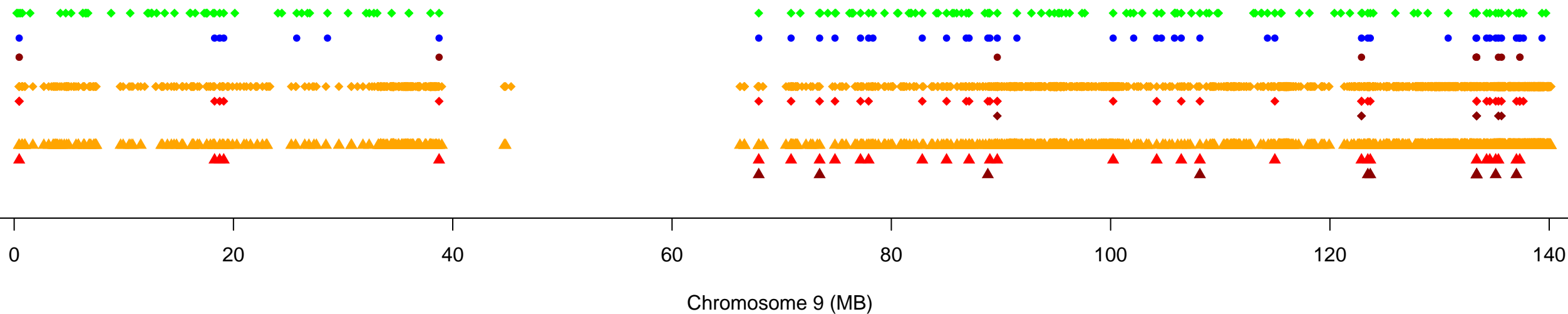

Supplement: Additional file 2 — Illustration of deletion clusters inferred by Algorithm 1, GASV and BreakDancer. Note that many deletions in close proximity may appear as a single dot, and the size of a dot is in general larger than the respective deletion. For some data sets, the computation of all maximal clusters was infeasible. This ZIP-archive contains a PDF file for each chromosome. [file 1471-2105-12-S9-S21-S2.zip › allChromosomesWithLegend/chr9WithLegend.pdf]

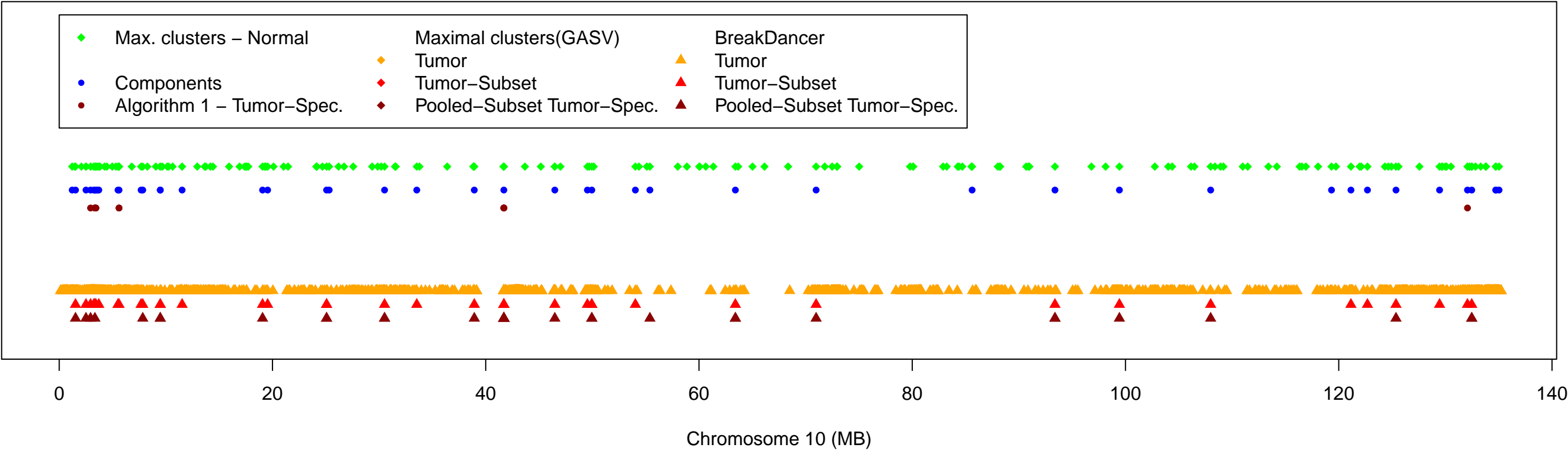

Supplement: Additional file 2 — Illustration of deletion clusters inferred by Algorithm 1, GASV and BreakDancer. Note that many deletions in close proximity may appear as a single dot, and the size of a dot is in general larger than the respective deletion. For some data sets, the computation of all maximal clusters was infeasible. This ZIP-archive contains a PDF file for each chromosome. [file 1471-2105-12-S9-S21-S2.zip › allChromosomesWithLegend/chr10WithLegend.pdf]

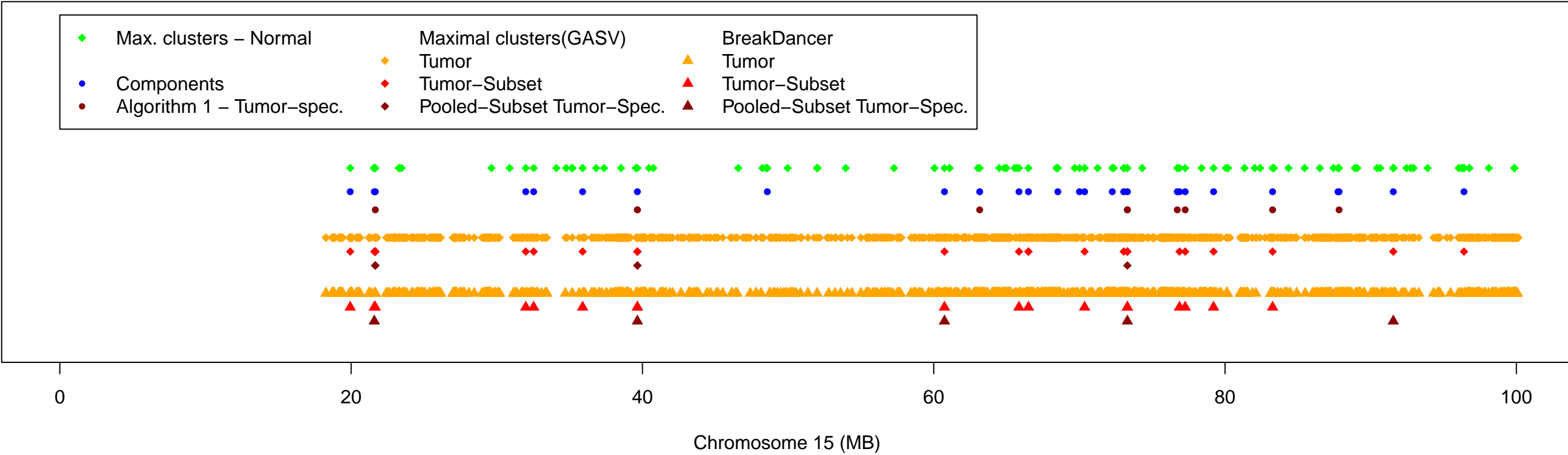

Supplement: Additional file 2 — Illustration of deletion clusters inferred by Algorithm 1, GASV and BreakDancer. Note that many deletions in close proximity may appear as a single dot, and the size of a dot is in general larger than the respective deletion. For some data sets, the computation of all maximal clusters was infeasible. This ZIP-archive contains a PDF file for each chromosome. [file 1471-2105-12-S9-S21-S2.zip › allChromosomesWithLegend/chr15WithLegend.pdf]

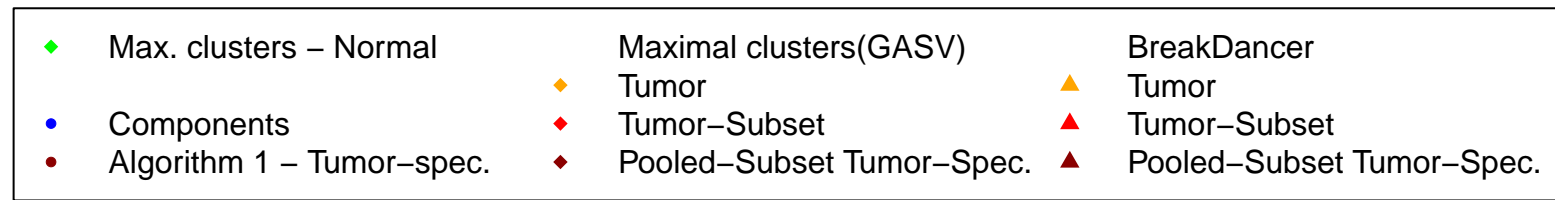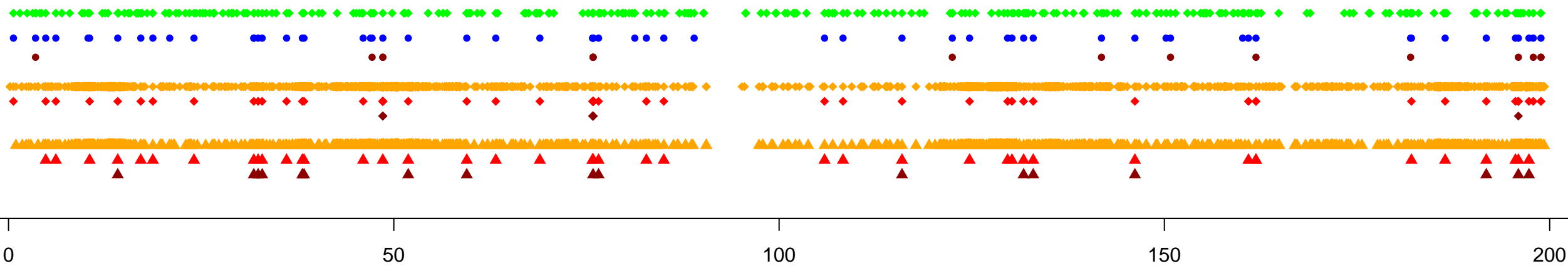

Chromosome 3 (MB)

Supplement: Additional file 2 — Illustration of deletion clusters inferred by Algorithm 1, GASV and BreakDancer. Note that many deletions in close proximity may appear as a single dot, and the size of a dot is in general larger than the respective deletion. For some data sets, the computation of all maximal clusters was infeasible. This ZIP-archive contains a PDF file for each chromosome. [file 1471-2105-12-S9-S21-S2.zip › allChromosomesWithLegend/chr3WithLegend.pdf]

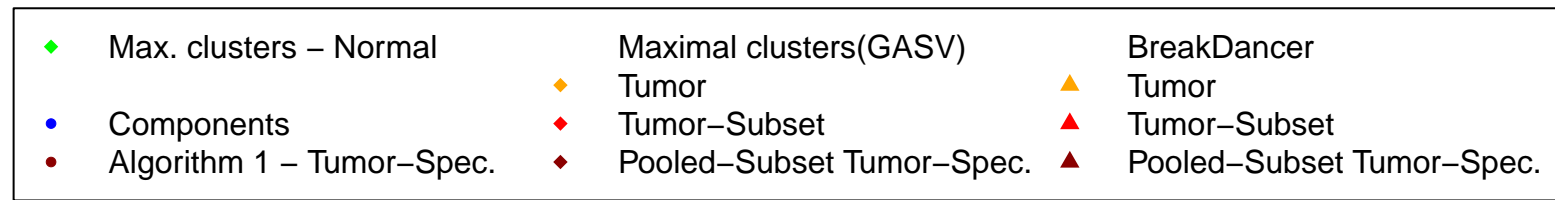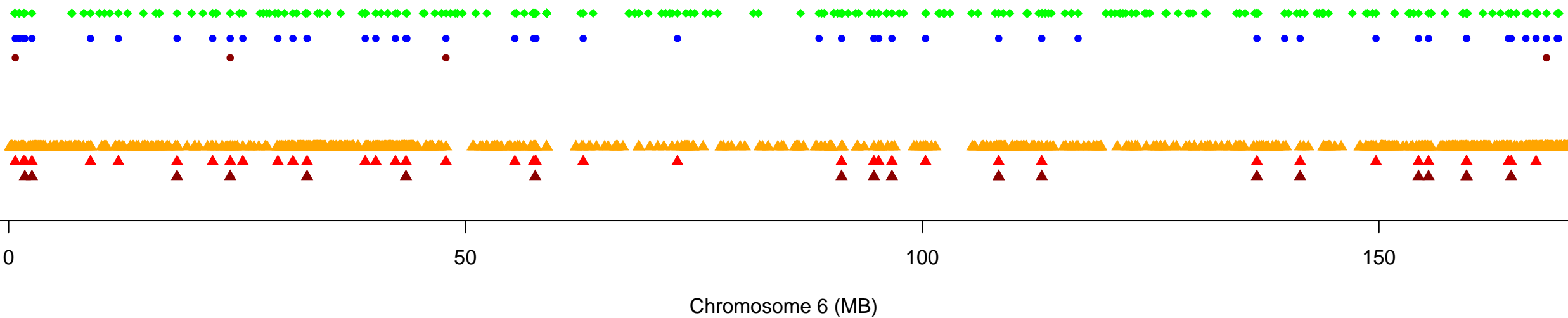

Supplement: Additional file 2 — Illustration of deletion clusters inferred by Algorithm 1, GASV and BreakDancer. Note that many deletions in close proximity may appear as a single dot, and the size of a dot is in general larger than the respective deletion. For some data sets, the computation of all maximal clusters was infeasible. This ZIP-archive contains a PDF file for each chromosome. [file 1471-2105-12-S9-S21-S2.zip › allChromosomesWithLegend/chr6WithLegend.pdf]
